# Cyclic increase in the ADAMTS1-L1CAM-EGFR axis promotes the EMT and cervical lymph node metastasis of oral squamous cell carcinoma

**DOI:** 10.1038/s41419-024-06452-9

**Published:** 2024-01-23

**Authors:** Ming-Hsien Chien, Yi-Chieh Yang, Kuo-Hao Ho, Yi-Fang Ding, Li-Hsin Chen, Wen-Kuan Chiu, Ji-Qing Chen, Min-Che Tung, Michael Hsiao, Wei-Jiunn Lee

**Affiliations:** 1 Graduate Institute of Clinical Medicine, College of Medicine, Taipei Medical University, Taipei, Taiwan; 2 TMU Research Center of Cancer Translational Medicine, Taipei Medical University, Taipei, Taiwan; 3 Pulmonary Research Center, Wan Fang Hospital, Taipei Medical University, Taipei, Taiwan; 4 Traditional Herbal Medicine Research Center, Taipei Medical University Hospital Taipei, Taipei, Taiwan; 5 Department of Medical Research, Tungs’ Taichung MetroHarbor Hospital, Taichung, Taiwan; 6 Graduate Institute of Medical Sciences, College of Medicine, Taipei Medical University, Taipei, Taiwan; 7 Department of Otolaryngology, Wan Fang Hospital, Taipei Medical University, Taipei, Taiwan; 8 Division of Plastic Surgery, Department of Surgery, Wan Fang Hospital, Taipei Medical University, Taipei, Taiwan; 9 Department of Surgery, School of Medicine, College of Surgery, Taipei Medical University, Taipei, Taiwan; 10 Department of Cancer Biology, Geisel School of Medicine at Dartmouth, Lebanon, NH USA; 11 Department of Surgery, Tungs’ Taichung Metro Harbor Hospital, Taichung, Taiwan; 12 Genomics Research Center, Academia Sinica, Taipei, Taiwan; 13 Department of Urology, School of Medicine, College of Medicine, Taipei Medical University, Taipei, Taiwan; 14 Department of Medical Education and Research, Wan Fang Hospital, Taipei Medical University, Taipei, Taiwan

**Keywords:** Oral cancer, Extracellular matrix, Epithelial-mesenchymal transition

## Abstract

The matrix metalloprotease A disintegrin and metalloprotease with thrombospondin motifs 1 (ADAMTS1) was reported to be involved in tumor progression in several cancer types, but its contributions appear discrepant. At present, the role of ADAMTS1 in oral squamous cell carcinoma (SCC; OSCC) remains unclear. Herein, The Cancer Genome Atlas (TCGA) database showed that ADAMTS1 transcripts were downregulated in head and neck SCC (HNSCC) tissues compared to normal tissues, but ADAMTS1 levels were correlated with poorer prognoses of HNSCC patients. In vitro, we observed that ADAMTS1 expression levels were correlated with the invasive abilities of four OSCC cell lines, HSC-3, SCC9, HSC-3M, and SAS. Knockdown of ADAMTS1 in OSCC cells led to a decrease and its overexpression led to an increase in cell-invasive abilities in vitro as well as tumor growth and lymph node (LN) metastasis in OSCC xenografts. Mechanistic investigations showed that the cyclic increase in ADAMTS1-L1 cell adhesion molecule (L1CAM) axis-mediated epidermal growth factor receptor (EGFR) activation led to exacerbation of the invasive abilities of OSCC cells via inducing epithelial-mesenchymal transition (EMT) progression. Clinical analyses revealed that ADAMTS1, L1CAM, and EGFR levels were all correlated with worse prognoses of HNSCC patients, and patients with ADAMTS1^high^/L1CAM^high^ or EGFR^high^ tumors had the shortest overall and disease-specific survival times. As to therapeutic aspects, we discovered that an edible plant-derived flavonoid, apigenin (API), drastically inhibited expression of the ADAMTS1-L1CAM-EGFR axis and reduced the ADAMTS1-triggered invasion and LN metastasis of OSCC cells in vitro and in vivo. Most importantly, API treatment significantly prolonged survival rates of xenograft mice with OSCC. In summary, ADAMTS1 may be a useful biomarker for predicting OSCC progression, and API potentially retarded OSCC progression by targeting the ADAMTS1-L1CAM-EGFR signaling pathway.

## Introduction

Oral squamous cell carcinoma (SCC; OSCC) is one of the major types of head and neck SCC (HNSCC), accounting for more than half of HNSCCs [1], the sixth most frequently diagnosed cancer globally [2]. Cervical lymph node (LN) metastasis in OSCC is a critical factor in terms of increased recurrence and disease-specific and overall death [3]. Currently, there are various diagnostic tools available to detect LN metastasis of OSCC such as ultrasound, magnetic resonance imaging, and computed tomographic scans, but their efficacy in detecting cervical LN metastasis is still being debated due to the presence of occult LN metastasis [4]. Treatment possibilities of recurrent and metastatic OSCC are limited, and no marked improvement in survival probability has so far been achieved with current treatment options [5]. Therefore, early detection and optimum treatment of cervical LN metastasis are absolutely necessary for better prognostic outcomes of OSCC.

Extracellular matrix (ECM) components are involved in various aspects of tumor biology, including metastatic events. For aberrant remodeling of the ECM in the tumor microenvironment, cancer cells rely on the proteolytic activity of ECM proteases to break down structural ECM proteins and subsequently move through tissue barriers [6, 7]. Metalloproteases belonging to the A disintegrin and metalloprotease with thrombospondin motifs (ADAMTS) family were implicated in ECM remodeling events manifested in cancer development and progression [8]. Nineteen ADAMTS proteases have so far been identified in humans, and the dysregulated expression of ADAMTS1 is the best characterized in cancer [9]. ADAMTS1 was reported to target specific substrates that exist in the ECM including modular proteoglycans (versican, aggrecan, and syndecan-4) [10, 11] collagen [12], and growth factors (amphiregulin, heparin-binding EGF (HB-EGF), and transforming growth factor (TGF)-β) [13]. Although roles for ADAMTS1 were suggested in promoting tumor invasion and metastasis in kidney, brain, and breast tumors [14–16], its role in proteoglycan proteolysis also has tumor-suppressive functions [17]. As to the role of ADAMTS1 in tumor types located on the head and neck, ADAMTS1 was shown to sequester vascular endothelial growth factor C (VEGF-C) and block the lymphangiogenesis and lymphatic metastasis of esophageal SCC [18]. To the present, the role of ADAMTS1 in OSCC progression remains elusive.

The aim of the present work was to identify the function and mechanism of ADAMTS1 in OSCC progression. An in silico analysis showed that ADAMTS1 expression levels were lower in HNSCC tissues compared to normal tissues, but were correlated with shorter survival times in HNSCC patients. In vitro and in vivo loss- and gain-of-function experiments revealed that ADAMTS1 is functionally involved in the invasion and LN metastasis of OSCC. Mechanistic studies revealed that the cyclic increase in the ADAMTS1-L1 cell adhesion molecule (L1CAM) axis and epidermal growth factor receptor (EGFR) activation led to exacerbation of the invasive abilities of OSCC cells via inducing epithelial-mesenchymal transition (EMT) progression. Combined expressions of high L1CAM or EGFR and high ADAMTS1 were correlated with the worst prognoses of HNSCC patients. Furthermore, apigenin (API) was identified as a potential inhibitor targeting the ADAMTS1-L1CAM-EGFR axis which significantly reversed ADAMTS1-induced invasion and cervical LN metastasis of OSCC cells in in vitro and in vivo models, respectively.

## Materials and methods

### Materials

API (A3145) and dimethyl sulfoxide (DMSO) were purchased from Sigma-Aldrich (St. Louis, MO, USA). Fetal bovine serum (FBS), antibiotics, molecular weight standards, trypsin-EDTA, and all medium additives were obtained from Life Technologies (Gaithersburg, MD, USA). A recombinant human L1CAM (777-NC) was purchased from R&D Systems (Minneapolis, MN, USA). Antibodies used in the Western blot and dot blot analyses are described here. Antibodies for phosphorylated (p-) and unphosphorylated forms of several kinases and EMT-related markers including EGFR (#3777 and #4267), Src (#2101), AKT (#9271 and #9272), extracellular signal-regulated kinase (ERK) (#4370 and #4695), signal transduction and activator of transcription 3 (Stat3) (#9145 and #9139), zonula occludens (ZO)-1 (#8193), E-cadherin (#3195), vimentin (#5741), and Snail (#3879) were obtained from Cell Signaling Technology (Danvers, MA, USA). An antibody specific for ADAMTS1 (AF5867) was purchased from R&D Systems, while those for L1CAM (sc514360) and TGF-β (sc130348) were from Santa Cruz Biotechnology (Santa Cruz, CA, USA). Moreover, Proteintech Group (Chicago, IL, USA) supplied the antibodies for GAPDH (60004-1-Ig) and α-tubulin (66031-1-Ig), and Sigma-Aldrich supplied the antibody for β-actin (A5441).

### Data collection from bioinformatics analyses

Clinical data and messenger (m)RNA sequencing of HNSCC in The Cancer Genome Atlas (TCGA) were obtained from the UCSC Xena database (https://xena.ucsc.edu/). OSCC patients were selected from TCGA HNSCC with tumor sites at the buccal mucosa, alveolar ridge, floor of the mouth, hard palate, oral cavity, and tongue. Gene expressions from paired tumor/normal tissues were compared by a paired *t-*test. Associations of ADAMTS1, L1CAM, and EGFR with patient’s overall survival (OS) and disease-specific survival (DSS) were evaluated by a log-rank test. For three-group comparisons, pairwise log-rank tests were performed. The expression cutoff to define the high- and low-expression groups was based on medium values.

### Cell lines and cell culture

The human OSCC cell lines of HSC-3, HSC-3M, SAS, and SCC9 cells were purchased from the Japanese Collection of Research Bioresources (JCRB) Cell Bank (Osaka, Japan). HSC-3 and HSC-3M cells were cultured in minimal essential medium (MEM, Gibco, Grand Island, NY, USA); SCC9 and SAS cells were maintained in Dulbecco’s modified Eagle medium/nutrient mixture F-12 (DMEM/F-12, Gibco), supplemented with 10% FBS and 100 U/mL of penicillin and streptomycin at 37 °C in a humidified atmosphere of 5% CO_2_.

### Establishment of gene knockdown and overexpression of OSCC cells

The pLEX-MCS-ADAMTS1 and pcDNA3.1-EGFR expression constructs were respectively obtained from Dr. T.C. Kuo and Dr. K.T. Hua (National Taiwan University, Taipei, Taiwan). pcDNA3.1-L1CAM was purchased from Addgene (Watertown, MA, USA). Short hairpin (sh)RNAs for ADAMTS1, L1CAM, and EGFR were purchased from the RNA Technology Platform and Gene Manipulation Core Facility at Academic Sinica (Taipei, Taiwan). To knockdown the indicated genes (ADAMTS1, L1CAM, and EGFR) or overexpress ADAMTS1, we produced lentiviral particles expressing shRNAs of the indicated genes or pLEX-MCS-ADAMTS1 to infect OSCC cells for 24 h according to protocols from our previous study [19]. To overexpress L1CAM or EGFR in OSCC cells, Lipofectamine 3000 Transfection Reagent (Invitrogen, Carlsbad, CA, USA) was used. Targeting sequences of the shRNAs were as follows: ADAMTS1 shRNA-1, 5′-CCACAGGAACTGGAAGCATAA-3′; ADAMTS1 shRNA-2, 5′-GCCTACATGATTACATCATTT-3′; L1CAM shRNA-1, 5′-GCTAACCTGAAGGTTAAAGAT-3′; L1CAM shRNA-2, 5′-GCCAATGCCTACATCTACGTT-3′; and EGFR shRNA, 5′-GCCAAGCCAAATGGCATCTTT-3′.

### Western blot assay

Total cell lysate extraction and protein concentration measurements were performed as previously described [20]. Appropriate quantities of protein (30~50 μg) were separated by sodium dodecylsulfate-polyacrylamide gel electrophoresis (SDS-PAGE) and transferred onto polyvinylidene difluoride (PVDF) membranes (Merck Millipore, Burlington, MA, USA). The membranes were then incubated with the indicated primary antibodies and horseradish peroxidase-conjugated secondary antibodies. Next, blots were washed with TBST washing buffer and visualized with the ECL Western blotting reagent (TOOLS, New Taipei City, Taiwan), and chemiluminescence was detected with the MultiGel-21 chemiluminescence imaging system (TOP BIO, New Taipei City, Taiwan).

### Transwell invasion assay

The in vitro invasive ability of OSCC cells was detected according to our previous study [21]. Briefly, 5 × 10^4^ ADAMTS1-manipulated OSCC cells were pretreated with or without API and plated in a Matrigel (BD Biosciences, Bedford, MA, USA)-coated top chamber (24-well insert; pore size, 8 μm; Corning Costar, Corning, NY, USA) containing serum-free medium. Medium supplemented with serum was used as a chemoattractant in the lower chamber. After 48 h of incubation, cells that had invaded to the bottom surface of the insert were fixed in 100% methanol and stained with crystal violet. The number of cells invading through the membrane was visualized and counted under a light microscope (200×, three random fields per well).

### Cell viability assay

ADAMTS1-manipulated OSCC cells and their respective control cells were seeded in 96-well plates (5 × 10^3^ cells/well) containing 200 μl of complete culture medium. After incubation of cells for different time points (24~96 h), cell viability was determined with a CellTiter 96 Aqueous One Solution Cell Proliferation Assay (MTS assay; Promega, Madison WI, USA) according to the manufacturer’s instructions. Data were collected from three replicates.

### Plate colony-forming assay

HSC-3 and HSC-3M OSCC cells expressing ADAMTS1-flag, ADAMTS1 shRNAs, or their respective control vector were seeded in six-well plates at a density of 10^3^ cells/well and incubated for 24 h. Next, the culture medium was changed every 2 days, and cells were fixed with 4% paraformaldehyde and stained with 0.1% crystal violet after 10 days’ incubation. Violet-stained colonies were photographed and quantified by dissolving the violet stain in 10% acetic acid. Plates were placed on a shaker for 15 min to destain all of the cells, and the destaining solution was measured by an enzyme-linked immunosorbent assay (ELISA) reader at 595 nm.

### In vivo orthotopic xenograft model

All animal experiments were performed according to a protocol approved by the Institutional Animal Care and Use Committee of Wan Fang Hospital (approval no.: WAN-LAC-112-04). For ADAMTS1 overexpression and knockdown experiments in an orthotopic xenograft mouse model, 6-week-old nonobese diabetic (NOD)-SCID male mice were anesthetized with isoflurane, and then the HSC-3-vector-luciferase, HSC-3-ADAMTS1-luciferase, HSC-3M-shCtrl-luciferase, or HSC-3M-shADAMTS1-luciferase-tagged cell lines (5 × 10^5^) were resuspended in 20 μl of a 1:1 mixture of phosphate-buffered saline (PBS) and Matrigel and submucosally injected in the floor of the mouth using a 30-gauge needle. Seven days after tumor cell implantation, mice were randomly assigned to the experimental and control groups according to the Xenogen IVIS spectrum bioluminescence imaging (BLI) results (Caliper; Xenogen, Alameda, CA, USA), and treatment was initiated according to similar mean tumor sizes in each group. Each treated mouse was intraperitoneally (IP) administered 3 mg/kg body weight API or the vehicle (10% DMSO in PBS) 5 days/week. Tumor growth and metastasis from each group were monitored weekly with this BLI system. At the end of the experiment, primary tumors and cervical LNs were harvested, fixed, sectioned, and stained with the indicated antibodies.

### Immunohistochemical (IHC) staining

IHC staining processes were as previously described [22]. In brief, paraffin-embedded tumor tissue sections were treated with xylene to remove the paraffin and incubated with a 0.3% H_2_O_2_ solution to block endogenous peroxidase activity that could interfere with the staining. Slides were then washed with PBS and incubated with a specific primary antibody for L1CAM (1:100) for 24 h at 4 °C. Following the PBS washing step, slides were developed with a VECTASTAIN ABC (avidin-biotin complex) peroxidase kit (Vector Laboratories, Burlingame, CA, USA) and DAB peroxidase substrate kit (Vector Laboratories), following the manufacturer’s instructions. Nuclei were counterstained with hematoxylin.

### Dot blot assay

ADAMTS1-manipulated OSCC cells at densities of 10^6^ cells/ml were seeded in a 10-cm Petri dish overnight. After cell attachment, cell culture medium was replaced with serum-free medium for another 48 h and harvested for dot blotting. Equal amounts of conditioned medium (200 μl) from OSCC cells were blotted onto nitrocellulose membranes by suction using GFE9600 dot-blotting equipment (Bio-East Technology, Taipei, Taiwan) at room temperature. Hybridization of the membrane with indicated antibodies (L1CAM and ADAMTS1) and detection of antibody-bound protein were performed according to the Western blot protocol.

### Co-Immunoprecipitation (Co-IP)

To detect physical interactions of LCAM, ADAMTS1, and EGFR, HEK293 cells overexpressing ADAMTS1, L1CAM, and EGFR were harvested with NETN lysis buffer (150 mM NaCl, 20 mM Tris-base, 1 mM EDTA, 0.5% NP-40). Cell lysates at 0.5 mg were incubated with an anti-EGFR, anti-LICAM, or anti-immunoglobulin G (IgG) antibody overnight at 4 °C followed by a 2-h incubation with 50 μl of immobilized protein A Sepharose beads. The protein complex was also washed with NETN buffer five times. Proteins collected from HEK293 cells were boiled in 5× sample dye and further analyzed by Western blotting.

### Gene set enrichment analysis (GSEA) and pathway enrichment analysis

To investigate signaling pathways associated with ADAMTS1, L1CAM, and EGFR expressions, a GSEA was conducted using Hallmark gene sets. Genes were ranked based on their Pearson correlation coefficients. A weighted Kolmogorov-Smirnov test was employed with 10^3^ permutations to calculate the normalized enrichment score (NES) and false discovery rate (FDR).

### Fluorescence microscopy

Approximately 2 × 10^4^ HSC-3 or HSC-3M cells, both with and without manipulated ADAMTS1 expression, were seeded in 8-well chamber slides (Millicell® EZ SLIDES, Millipore) and allowed to incubate for 16 hours. Subsequently, the cells were fixed with a 4% formaldehyde solution in PBS. Following fixation, the cells were stained using Phalloidin CruzFluor™ 594 Conjugate (1:1000) to visualize the cytoplasmic distribution of F-actin (depicted in red) and counterstained with Hoechst (1:500) for nuclear staining (displayed in blue). Cell imaging was conducted using a Leica Stellaris 8 Confocal Microscope at a magnification of 63x, located in the Core Facility Center at Taipei Medical University.

### Statistical analysis

Values are presented as the mean ± standard deviation (SD). Statistical analyses were performed using SPSS vers. 20 (SPSS, Chicago, IL, USA), and quantified data were analyzed using GraphPad Prism 7 (GraphPad Software, San Diego, CA, USA). Differences between two groups were analyzed using Student’s *t*-test.

## Results

### ADAMTS1 expression correlates with poor prognoses in HNSCC patients and promotes an invasive phenotype of OSCC cells

**Fig. 1:**
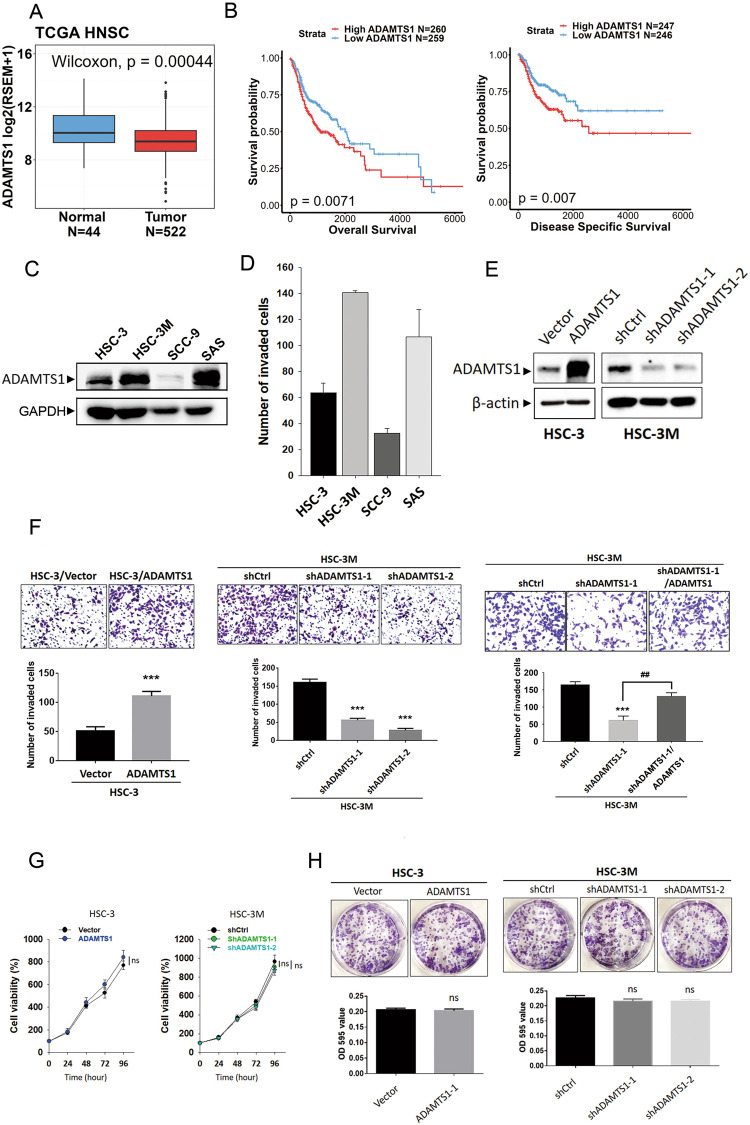
ADAMTS1 expression promotes invasion of oral squamous cell carcinoma (SCC; OSCC) cells and correlates with poor survival outcomes in patients with head and neck SCC (HNSCC). **A** *ADAMTS1* transcripts in normal and HNSCC tissues were analyzed using data from TCGA. Statistical significance was analyzed by a Wilcoxon signed-rank test. **B** Kaplan–Meier analysis of overall survival (OS) and disease-specific survival (DSS) rates in patients with HNSCC presenting with high or low ADAMTS1 expression using data from TCGA. **C** Endogenous protein levels of ADAMTS1 in OSCC cell lines (HSC-3, HSC-3M, SCC9, and SAS) were detected by a Western blot analysis. **D** In vitro invasive abilities of OSCC cell lines were analyzed by a Matrigel-invasion assay. **E** Western blot analysis of ADAMTS1 expressions in HSC-3 (left) and HSC-3M (right) cells respectively expressing ADAMTS1-flag and ADAMTS1 shRNAs. **F** Invasive abilities of HSC-3 and HSC-3M cells were determined by a Matrigel-invasion assay after infecting cells with a lentivirus carrying ADAMTS1-flag, ADAMTS1 shRNAs, ADAMTS1 shRNA co-expressing ADAMTS1-flag, or their respective control vectors. Upper panel: representative photomicrographs (200×). Lower panel: quantitative values from counting of invaded cells presented as the mean ± SD of three independent experiments. ***p < 0.001, compared to the respective control groups. ^##^*p* < 0.01, compared to ADAMTS1 shRNA-infected only group. **G**, **H** Proliferation rates and colony-formation capacities of ADAMTS1-manipulated HSC-3 and HSC-3M cells were respectively determined using MTS and colony-forming assays. ADAMTS1 expression had no obvious effect on cell proliferation rates during 24–96 h (**G**) or the colony-formation capacity during 7‒10 days (**H**). ns not significant.

We first analyzed correlations of ATAMTS1 expression levels and their prognostic significance in head and neck cancer, by examining cases of HNSCC from TCGA dataset. We observed that ADAMTS1 expression was significantly lower in HNSCC tissues compared to noncancerous tissues (Fig. 1A). The OSCC population (tumors that occurred on the buccal mucosa, alveolar ridge, floor of mouth, hard palate, oral cavity, and oral tongue) was further selected from the HNSCC cohort, and we also observed a similar phenomenon (Supplementary Fig. 1). Despite there being lower ADAMTS1 expression in tumor tissues, a Kaplan–Meier plot showed that HNSCC patients from TCGA with ADAMTS1^high^ tumors had shorter OS and DSS times compared to those with ADAMTS1^low^ tumors (Fig. 1B). After analyzing the clinical significance of ADAMTS1, we further investigated the effect of ADAMTS1 expression on cell behaviors of OSCC cells including SAS, SCC9, HSC-3, and its highly metastatic subline, HSC-3M. First, we observed HSC-3M and SAS cells expressing relatively high ADAMTS1 levels (Fig. 1C) and also exhibiting relatively high invasive abilities (Fig. 1D) among these OSCC cells. Next, we determined whether ADAMTS1 modulates the cell-invasive ability through overexpression and knockdown of ADAMTS1 by lentiviral-based approaches in HSC-3 and HSC-3M cells (Fig. 1E). Overexpressing ADAMTS1 in HSC-3 cells significantly increased the cell-invasive abilities while knocking-down ADAMTS1 by two ADAMTS1-specific shRNAs significantly suppressed HSC-3M cell invasion (Fig. 1F, left and middle panels). To confirm the dependence of ADAMTS1 in regulating cell invasion and to eliminate potential off-target effects of shRNA, we reintroduced ADAMTS1 into ADAMTS1-depleted HSC-3M cells and assessed their invasive capabilities. ADAMTS1 overexpression significantly reversed the decrease in invasive ability induced by ADAMTS1 knockdown (Fig. 1F, right panel). In addition, depletion of ADAMTS1 also decreased the invasive ability of SAS cells (Supplementary Fig. 2). In contrast to the cell-invasive ability, we found no significant changes in cell viability (Fig. 1G) or cell clonogenicity (Fig. 1H) between the control and ADAMTS1-manipulated groups, suggesting that none of the results from the invasion assay was influenced by possible differences in the cell proliferation rate. Taken together, the above results imply that the ECM turnover regulator, ADAMTS1, plays an oncogenic role through inducing invasion of OSCC cells.

### ADAMTS1 promotes tumorigenicity and cervical LN metastasis in OSCC orthotopic graft models

**Fig. 2:**
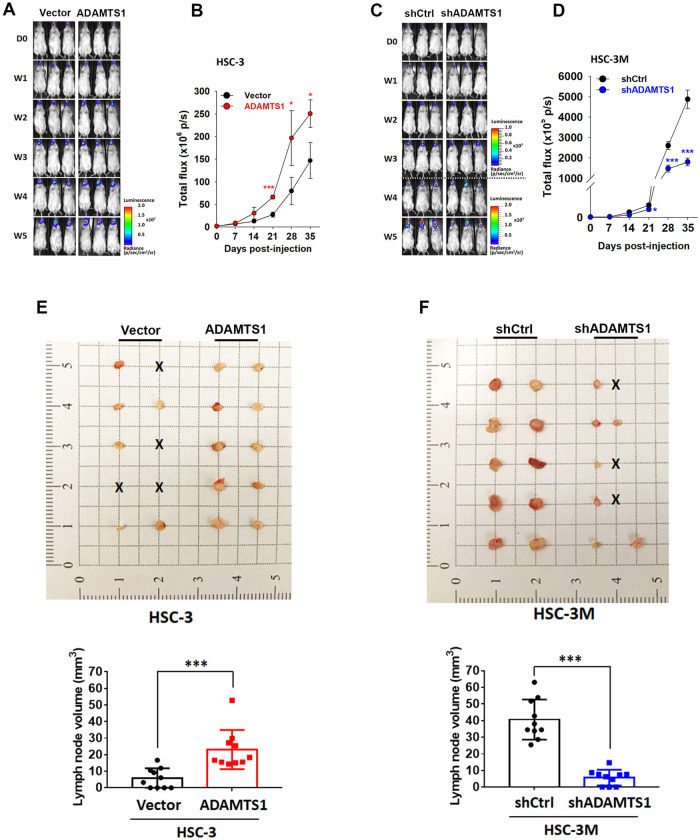
ADAMTS1 expression accelerates tumor growth and cervical lymph node (LN) metastasis of oral squamous cell carcinoma (OSCC) in orthotopic mouse models. Male NOD/SCID mice were orthotopically injected with luciferase-tagged and ADAMTS1-overexpressing HSC-3 cells or ADAMTS1-knockdown HSC-3M cells. **A**, **C** Whole-body bioluminescence imaging was conducted each week for 5 weeks after injecting ADAMTS1-manipulated cells into mice. **B**, **D** Quantitative analysis of Xenogen imaging signal intensity (photons/s/cm^2^/sr) every week. *p < 0.05, ***p < 0.001, compared to the control group. Occurrence of cervical LN metastasis in NOD/SCID mice implanted with HSC-3/ADAMTS1 (**E**), HSC-3M/shADAMTS1 (**F**), or their respective control cells. The appearance, number and volume of cervical LNs were photographed, enumerated, and measured after removal. Data are presented as the mean ± SD. ***p < 0.001 compared to the control group.

We next explored the in vivo effects of ADAMTS1 on OSCC progression through establishing an orthotopic OSCC-bearing model by transplanting luciferase (Luc)-tagged cells, HSC-3-vector-Luc, HSC-3M-shCtrl-Luc, HSC-3-ADAMTS1-Luc, or HSC-3M-shADAMTS1-Luc, into NOD-SCID mice. Tumor progression was monitored weekly by bioluminescence imaging (Fig. 2A, C). We observed that control HSC-3 cells (HSC-3/vector-Luc) orthotopically injected into NOD-SCID mice developed smaller tumors than did HSC-3/ADAMTS1-Luc cells injected into mice (Fig. 2B). In contrast, the tumorigenic ability was suppressed in ADAMTS1-depleted HSC-3M cells (Fig. 2D). At the end of the experiment, most mice had spontaneously developed cervical LN metastasis within 35 days after cancer cell injection, and frequencies of LN metastasis were higher and lower in mice respectively injected with HSC-3/ADAMTS1 and HSC-3M/shADAMTS1 cells compared to their respective control groups (Fig. 2E, F, upper panel). In addition, the mean volume of metastatic LNs in HSC-3/ADAMTS1 and HSC-3M/shADAMTS1 mice significantly increased and decreased compared with those in HSC-3/vector and HSC-3M/shCtrl mice, respectively (Fig. 2E, F, lower panel). Taken together, these results revealed that ADAMTS1 may promote progression of OSCC in vivo.

### L1CAM plays a critical role in the ADAMTS1-modulated invasive ability of OSCC cells

**Fig. 3:**
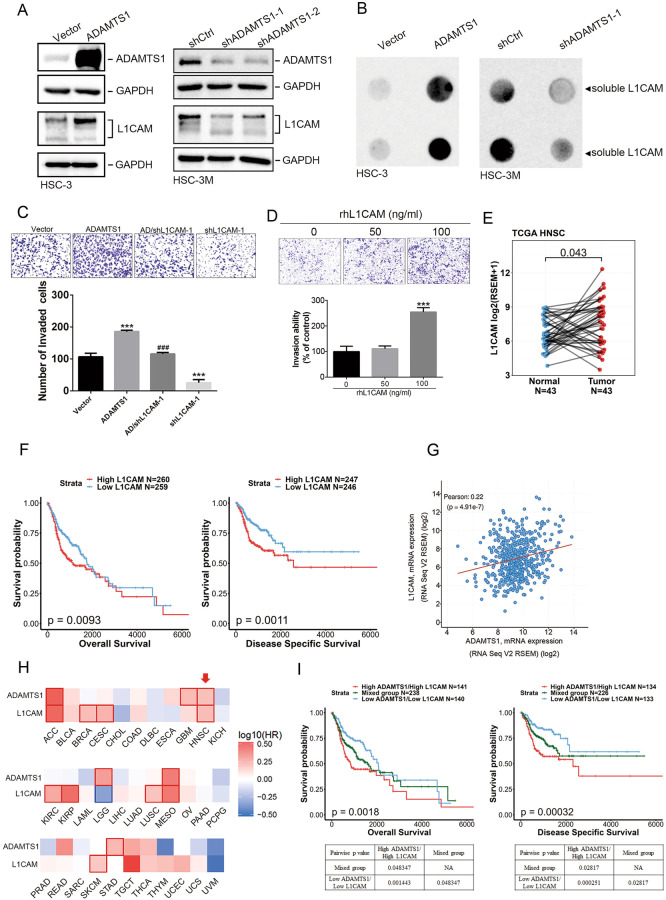
L1CAM is critical in ADAMTS1-modulated invasive ability of oral squamous cell carcinoma (SCC; OSCC) cells and was correlated with poor prognoses in patients with head and neck SCC (HNSCC). **A**, **B** HSC-3 or HSC-3M cells expressed ADAMTS1-flag, shADAMTS1, or their respective control as indicated. Cell lysates and conditioned media were collected to detect endogenous ADAMTS1 or L1CAM protein levels (**A**) and secreted soluble L1CAM (**B**), respectively using western blotting and Dot-blotting assays. **C** A L1CAM shRNA was transfected into ADAMTS1-overexpressing HSC-3 cells as indicated and subjected to Matrigel-invasion assays. **D** HSC-3 cells were treated with indicated concentrations of rhL1CAM for 24 h and subjected to Matrigel-invasion assays. **C**, **D** Multiples of differences are presented as the mean ± SD of three independent experiments. ***p < 0.001, compared to the control group; ^###^*p* < 0.001, compared to the ADAMTS1-overexpressing only group. **E** L1CAM expression was analyzed in 43 matched HNSCC tissues and their corresponding normal tissues using data from TCGA. **F** Kaplan–Meier analysis of overall survival (OS) and disease-specific survival (DSS) rates in patients with HNSCC presenting with high or low L1CAM expression using data from TCGA. **G** Correlation analysis of TCGA HNSCC databases (TCGA, PanCancer Atlas) using cBioPortal which revealed a positive correlation between expressions of ADAMTS1 and L1CAM. **H** Survival heat map showing the prognostic impacts of ADAMTS1 and L1CAM on 33 different cancer types according to the GEPIA2 database. HR hazard ratio. **I** Combined high ADAMTS1 expression and high L1CAM expression were correlated with the worst OS and DSS in patients with HNSCC compared to patients with other expression statuses of ADAMTS1 and L1CAM.

L1CAM is a glycoprotein that occurs in the ECM and is associated with cancer development, metastases, and poor prognoses. L1CAM-mediated cancer progression can be induced by proteolytic cleavage-products of full-length L1CAM (L1CAM-FL) by family members of a disintegrin and metalloproteinase (ADAM) such as ADAM10 and ADAM17 [23]. For example, L1CAM-FL was cleaved by ADAM10 to generate a soluble 180-kDa L1CAM (L1-180) [24]. ADAMTS1 and ADAM10 belong to the ADAM family and have both disintegrin and zinc metalloprotease domains, which implies that they might have functions related to the ECM and adhesion molecules by a similar approach [25]. Herein, we observed that overexpression and knockdown of ADAMTS1 respectively promoted and suppressed expression of L1-180 in HSC-3 and HSC-3M cells (Fig. 3A). Moreover, secretion of soluble L1CAM also respectively increased and decreased in both cell lines (Fig. 3B). In addition, ADAMTS1-knockdown-induced downregulation of L1CAM was also observed in SAS cells (Supplementary Fig. 3). Functionally, L1CAM-knockdown significantly attenuated the cell-invasive abilities of HSC-3 cells and also reversed their ADAMTS1-promoted invasive ability (Fig. 3C). In contrast, treatment of HSC-3 cells with recombinant human (rh)L1CAM significantly promoted the invasion of cells (Fig. 3D). In the clinic, we observed significantly higher L1CAM levels in tumors compared to adjacent normal tissues, regardless of whether in HNSCC (Fig. 3E) or OSCC (Supplementary Fig. 4) cohorts from TCGA database, and high L1CAM in tumors was correlated with shorter OS and DSS times in HNSCC patients (Fig. 3F). In addition, we used the cBioPortal platform to analyze HNSCC samples from TCGA and found that ADAMTS1 expression was significantly correlated with L1CAM expression (Fig. 3G). To better understand the prognostic impacts of ADAMTS1 and L1CAM on cancers, survival maps from the GEPIA2 database revealed that both genes were correlated with poor prognosis in only three of the 33 analyzed tumor types, namely adrenocortical carcinoma (ACC), mesotheliomas (MESO), and HNSCC (Fig. 3H). Moreover, Kaplan-Meier analyses using the same TCGA database described above showed that HNSCC patients with ADAMTS1^high^/L1CAM^high^ tumors had the shortest OS and DSS times compared to the ADAMTS1^high^/L1CAM^low^, ADAMTS1^low^/L1CAM^high^, and ADAMTS1^low^/L1CAM^low^ groups (Fig. 3I). Taken together, these results revealed that L1CAM is vital in the ADAMTS1-modulated invasive phenotype of OSCC cells, and the ADAMTS1-L1CAM axis may be a critical event in promoting HNSCC progression.

### Activation of EGFR/erbB1 signals is involved in ADAMTS1-L1CAM axis-promoted invasion of OSCC cells

**Fig. 4:**
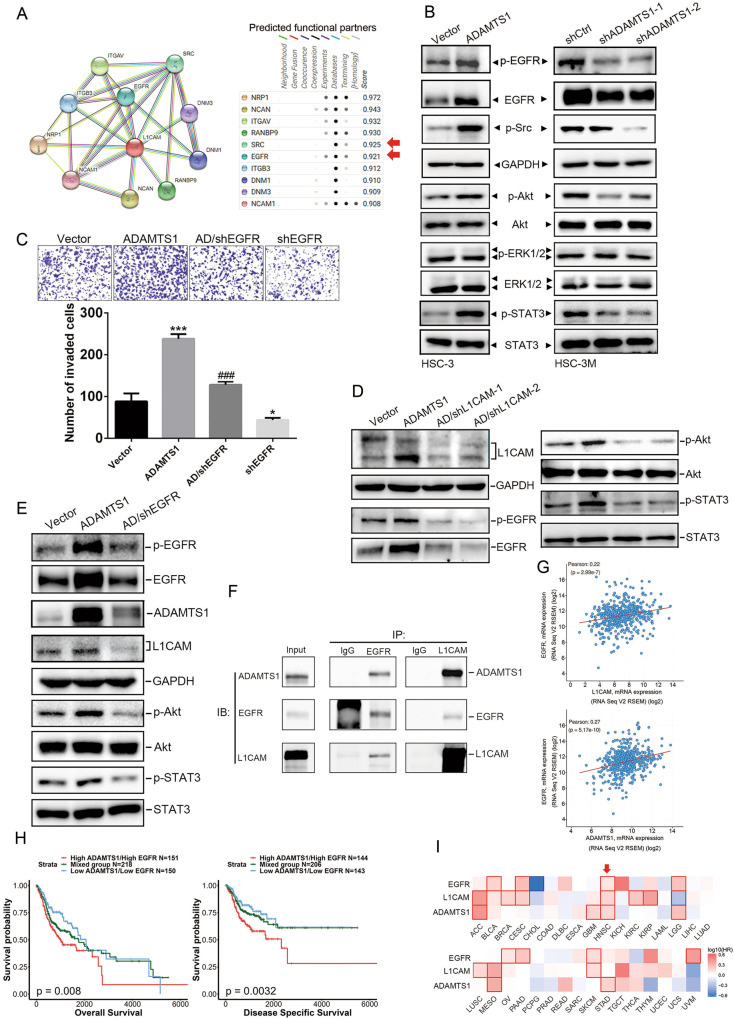
The ADAMTS1-L1CAM axis activates epidermal growth factor receptor (EGFR) signaling cascades to promote invasion of oral squamous cell carcinoma (SCC; OSCC) cells. **A** L1CAM protein-protein interaction network of 10 differentially expressed genes from the STRING database. **B** Detection of phosphorylation of the EGFR and its downstream signaling cascades by Western blotting after respective overexpression (left panel) and knockdown (right panel) of ADAMTS1 in HSC-3 and HSC-3M cells. **C** EGFR shRNA was transfected into ADAMTS1-overexpressing HSC-3 cells as indicated and subjected to Matrigel-invasion assays. Multiples of differences are presented as the mean ± SD of three independent experiments. *p < 0.05, ***p < 0.001, compared to the control group; ^###^*p* < 0.001, compared to the ADAMTS1-overexpressing only group. shRNAs specific for L1CAM (**D**) and EGFR (**E**) were transfected into ADAMTS1-overexpressing HSC-3 cells as indicated, and cell lysates were collected to detect the expressions or phosphorylation of ADAMTS1, L1CAM, EGFR, Akt, and Stat3. **F** The immunocomplex was precipitated from cell lysates of HEK293 cells overexpressing ADAMTS1, L1CAM, and EGFR with a L1CAM or EGFR antibody and analyzed by Western blotting to detect associations of EGFR, ADAMTS1, and L1CAM. The input was 10% whole-cell lysates. **G** Correlation analysis of EGFR with ADAMTS1 or L1CAM in head and neck SCC (HNSCC) tissues from TCGA using cBioPortal. **H** Combined high ADAMTS1 expression and high EGFR expression were correlated with the worst OS and DSS in patients with HNSCC compared to patients with other expression statuses of ADAMTS1 and L1CAM. **I** Survival heat map from the GEPIA2 database shows the poor prognostic impacts of ADAMTS1, L1CAM, and EGFR on HNSCC among 33 different cancer types. HR hazard ratio.

To further investigate the possible mechanisms participating in the ADAMTS1-L1CAM axis-mediated invasive ability of OSCC cells, we dissected interacting neighbors of L1CAM using the STRING database (Fig. 4A, left panel). We found that Src and EGFR were two of the top 10 interactors (Fig. 4A, right panel), and Src was reported to play a role in EGFR-mediated HNSCC cell invasion [26]. Herein, we observed that ADAMTS1 overexpression in HSC-3 cells triggered phosphorylation of EGFR (Tyr 1068) and Src (Tyr 416) and their downstream signals including Akt and Stat3 (Fig. 4B, left panel). In contrast, ADAMTS1-knockdown in HSC-3M cells was shown to suppress activation of EGFR, Src, Akt, and Stat3 (Fig. 4B, right panel). To our surprise, manipulation of ADAMTS1 in OSCC cells influenced the phosphorylation and also the expression of EGFR (Fig. 4B). Suppression of p-EGFR and EGFR was also observed in ADAMTS1-depleted SAS cells (Supplementary Fig. 5). Functionally, EGFR-knockdown significantly decreased the invasive ability and dramatically reversed promotion of invasion induced by ADAMTS1 overexpression in HSC-3 cells (Fig. 4C), suggesting the dependence on EGFR of the ADAMTS1-regulated cell-invasive ability.

Next, to dissect the relationship between L1CAM and the EGFR pathway modulated by ADAMTS1, we first knocked down L1CAM by L1CAM-specific shRNAs in HSC-3/ADAMTS1 cells. Figure 4D shows that L1CAM-knockdown dominantly reversed ADAMTS1 overexpression-induced upregulation of p-EGFR, EGFR, p-Akt, and p-Stat3. These results suggest that EGFR signaling may be triggered by the ADAMTS1-L1CAM axis to promote invasion by OSCC cells. In addition to the ADAMTS1-L1CAM-EGFR axis in regulating OSCC progression, we further observed that EGFR-knockdown also reversed ADAMTS1 overexpression-induced activation of EGFR signaling and upregulation of ADAMTS1, L1CAM, and EGFR in HSC-3 cells (Fig. 4E). Taken together, these data suggest that EGFR might exert feedback regulation on ADAMTS1 expression in OSCC cells. It was previously shown that human L1CAM-mediated homophilic cell interactions of EGFR-activated EGFR in *Drosophila* S2 cells [27]. In human embryonic HEK293 kidney cells, interaction of L1CAM and EGFR was also demonstrated [28]. Interaction of L1CAM and the erbB3 receptor was reported to enhance the response of erbB3 to the EGF-like factors, neuregulins, in MCF-7 breast cancer cells [28]. Herein, we co-transfected constructs respectively expressing ADAMTS1, L1CAM, and EGFR into HEK293 cells and found that both L1CAM and ADAMTS1 were present in the immunocomplex precipitated by an EGFR-specific antibody. We also observed that ADAMTS1 and EGFR were present in the immunocomplex precipitated by a L1CAM-specific antibody (Fig. 4F). These results implied that these three proteins physically interact when expressed in heterologous systems. In vivo, we also observed a significant reversal of increased tumor growth and cervical LN metastasis in ADAMTS1-overexpressed HSC-3 cells when these cells were engineered to express shRNA targeting L1CAM or EGFR (Supplementary Fig. 6). In the clinic, we found that EGFR expression was significantly correlated with ADAMTS1 and L1CAM expressions in HNSCC tissues from TCGA (Fig. 4G). Kaplan–Meier analyses using the same TCGA database revealed that HNSCC patients with ADAMTS1^high^/EGFR^high^ tumors had the worst prognosis (Fig. 4H). Survival maps from the GEPIA2 database showed that among the 33 analyzed cancer types, increased expressions of ADAMTS1, L1CAM, and EGFR were only significantly associated with poor survival in HNSCC (Fig. 4I), implying the critical and specific role of the ADAMTS1-L1CAM-EGFR axis in HNSCC progression.

### The ADAMTS1-L1CAM-EGFR axis promotes the invasive ability of OSCC cells via triggering the EMT

**Fig. 5:**
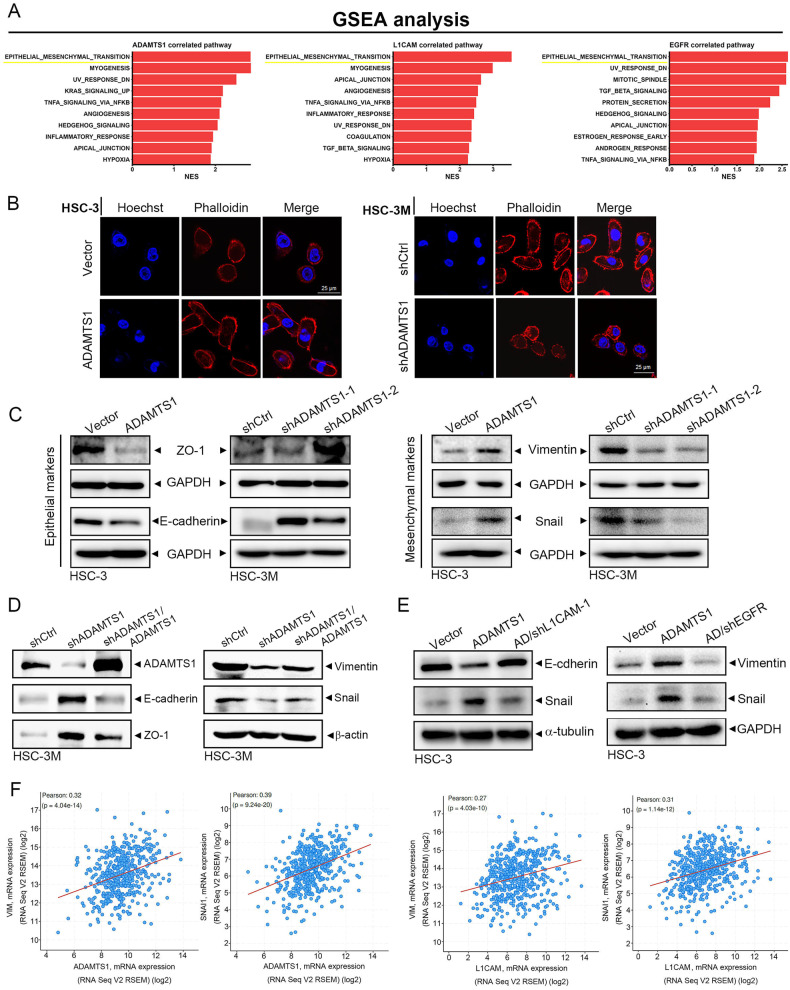
The ADAMTS1-L1CAM-EGFR axis triggers the epithelial-mesenchymal transition (EMT) in oral squamous cell carcinoma (SCC; OSCC) cells. **A** The top signaling associated with high ADAMTS1, L1CAM, and EGFR expressions is illustrated using a horizontal bar plot. To perform the GSEA, RNA sequencing data from TCGA head and neck SCC (HNSCC) were utilized, and the Hallmark pathway database was used to assess pathways enriched in patients with high expressions of ADAMTS1, L1CAM, or EGFR. Results showed the top ten signaling pathways with the highest normalized enrichment scores (NESs). **B** Detection of cellular morphology changes by phalloidin staining (in red) after respective overexpression and knockdown of ADAMTS1 in HSC-3 and HSC-3M cells. Nuclei were counterstained with Hoechst (in blue). Scare bar, 25 μm. **C** Detection of expressions of EMT-related signaling cascades including epithelial markers (zonula occludens (ZO)-1 and E-cadherin) and mesenchymal markers (vimentin and Snail) by Western blotting after respective overexpression and knockdown of ADAMTS1 in HSC-3 and HSC-3M cells. **D** HSC-3M cells expressing ADAMTS1 shRNA with or without co-expressing ADAMTS1-flag as indicated. Expressions of EMT-related markers were determined by western blotting. **E** An shRNA targeting L1CAM (left panel) or EGFR (right panel) was transfected into ADAMTS1-overexpressing HSC-3 cells as indicated, and cell lysates were collected to detect expressions of EMT-related markers. **F** Correlation analysis of TCGA HNSCC database (TCGA, PanCancer Atlas) using cBioPortal revealed positive correlations between ADAMTS1 or L1CAM and mRNA levels of indicated EMT-related genes.

To decipher the mechanism underlying the ADAMTS1-L1CAM-EGFR axis-modulated invasive phenotype of OSCC cells, a GSEA based on TCGA-HNSCC dataset was performed. We identified that the EMT is the top Hallmark gene set among the ADAMTS1-high, L1CAM-high, and EGFR-high group (Fig. 5A). The EMT is defined as a process where epithelial cells take on characteristics of mesenchymal cells and play vital roles in LN metastasis of OSCC [29]. Indeed, in HSC-3 and HSC-3M cells, we observed a distinct morphological transformation driven by ADAMTS1. In comparison to the control HSC-3 cells, which typically display a rounded shape, the ADAMTS1-overexpressed cells exhibited an elongated morphology with pronounced F-actin staining (Fig. 5B, left panel). Conversely, we also noted significant alterations in cell morphology among the HSC-3M cells following ADAMTS1 knockdown, where they adapted into a cuboidal shape, accompanied by a reduction in F-actin fibers (Fig. 5B, right panel). The morphological changes induced by ADAMTS1, which include alterations in cell shape and F-actin fiber distribution, are indicative of an enhanced front-rear polarity and are characteristic of a process known as EMT [30]. In addition, ADAMTS1 overexpression in HSC-3 cells led to EMT promotion, as evidenced by downregulation of the epithelial markers, ZO-1 and E-cadherin, and upregulation of the mesenchymal markers, vimentin and Snail. In contrast, upregulation of epithelial markers and downregulation of mesenchymal markers were observed in HSC-3M cells with ADAMTS1 depletion (Fig. 5C). We reintroduced ADAMTS1 into ADAMTS1-depleted HSC-3M cells and observed that overexpression of ADAMTS1 reversed the increase in ZO-1 and E-cadherin, as well as the decrease in vimentin and snail caused by ADAMTS1 knockdown (Fig. 5D). Figure 5E shows that knockdown of L1CAM or EGFR could reverse ADAMTS1 overexpression-induced increases in mesenchymal markers (vimentin and Snail) and the decrease in an epithelial marker (E-cadherin) in HSC-3 cells, suggesting that the ADAMTS1-L1CAM-EGFR axis promotes the invasive ability of OSCC cells via triggering the EMT. Consistently, we also observed positive correlations of ADAMTS1 or L1CAM expressions with mesenchymal markers of vimentin and Snail (SNAI1) in HNSCC tissues from TCGA dataset (Fig. 5F).

### Significant anti-invasion effects of API via targeting ADAMTS1 in OSCC cells

**Fig. 6:**
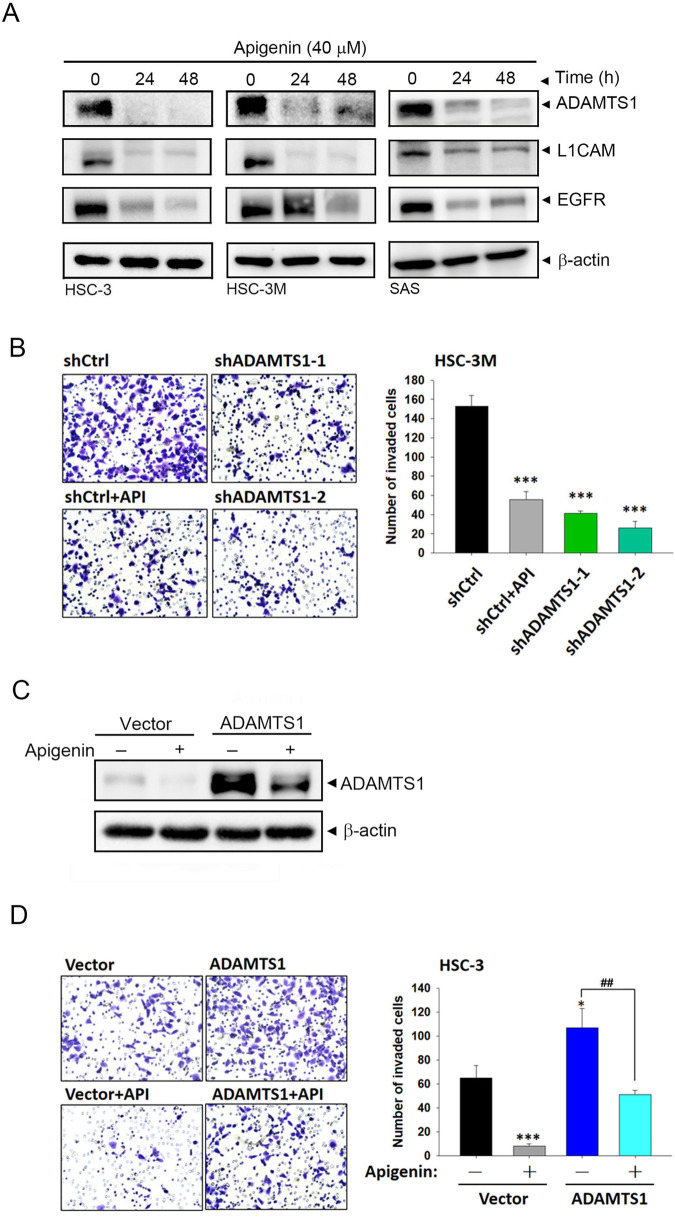
Targeting ADAMTS1 by apigenin (API) resulted in suppression of the invasion of oral squamous cell carcinoma (OSCC) cells. **A** HSC-3, HSC-3M, and SAS cells were treated with API at 40 μM for different durations, and ADAMTS1, L1CAM, and EGFR expressions were evaluated by Western blotting. **B** HSC-3M cells were treated with API or infected with ADAMTS1 shRNAs for 24 h, and cell-invasive abilities were measured by a Matrigel-invasion assay. **C**, **D** HSC-3 cells were transiently transfected with a vector control or ADAMTS1-flag followed by API or vehicle treatment for an additional 24 h. ADAMTS1 expression and invasive ability in cells were respectively detected by Western blotting (**C**) and Matrigel-invasion assays (**D**). **B**, **D** Representative photographs of invaded cells (left panel) and quantification of those cells (right panel). Data are presented as the mean ± SD of three independent experiments, *p < 0.05, ***p < 0.001 vs. control cells and ^##^*p* < 0.01, vs. ADAMTS1-overexpressing only cells.

After elucidating the oncogenic role and related mechanisms of ADAMTS1 in OSCC, we next tried to search for potential inhibitors of ADAMTS1. Previously, API, one of the most common flavonoids in vegetables, fruits, and Chinese medicinal herbs, was shown to inhibit expressions of ADAMTS4 and ADAMTS5 in articular chondrocytes [31]. Because ADAMTS1, ADAMTS4, and ADAMTS5 belong to the same ADAMTS subfamily [32], whether API has an inhibitory effect on ADAMTS1 in OSCC was determined by a Western blot analysis. Herein, we actually observed downregulation of ADAMTS1, L1CAM, and EGFR by API treatment in three OSCC cell lines (HSC-3, HSC-3M, and SAS) (Fig. 6A), suggesting that suppression of the ADAMTS1-L1CAM-EGFR axis by API generally occurs in OSCC cells. Functionally, we observed that treatment of HSC-3M cells with API showed similar anti-invasion effects caused by ADAMTS1 depletion (Fig. 6B). Moreover, ADAMTS1 upregulation and promotion of invasion caused by ADAMTS1 overexpression were all significantly reversed under API treatment of HSC-3 cells (Fig. 6C, D). These results suggested that API can abolish the cell-invasive ability of OSCC cells via targeting ADAMTS1.

### Significant anticancer progression effects of API via targeting ADAMTS1 in OSCC orthotopic graft models

**Fig. 7:**
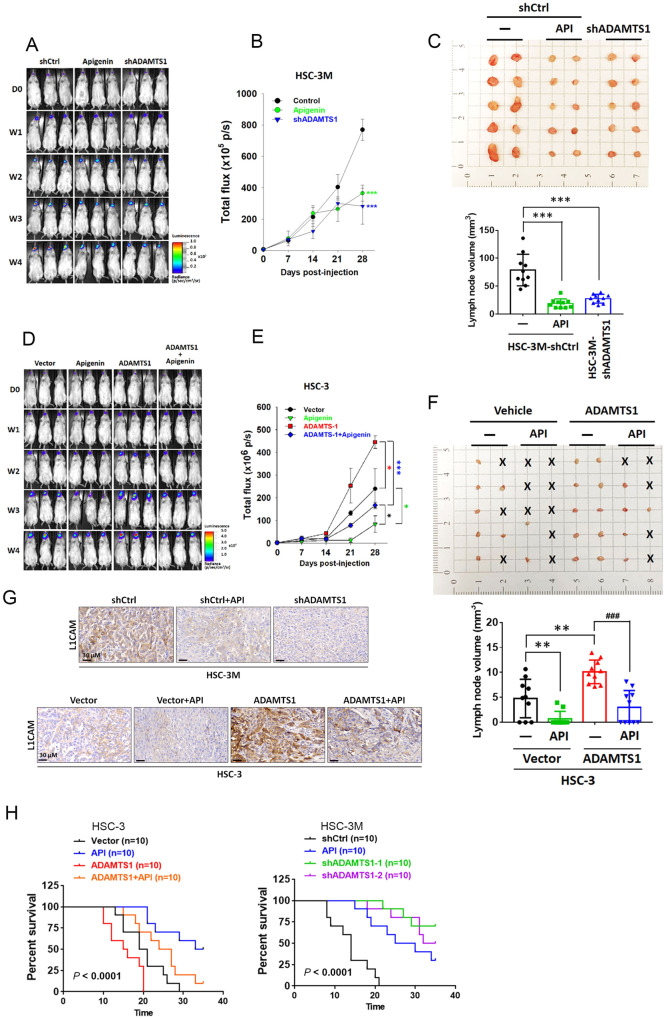
Targeting ADAMTS1 by apigenin (API) suppresses growth and lymph node (LN) metastasis of oral squamous cell carcinoma (OSCC) cells in orthotopic mouse models. Male NOD/SCID mice were orthotopically implanted with luciferase-tagged and ADAMTS1-depleted HSC-3M cells, ADAMTS1-overexpressing HSC-3 cells, or their respective control cells. At 7 days after injection of cells, mice were intraperitoneal (IP) administered API (3 mg/kg body weight) or the vehicle as indicated for 5 days/week. (**A** and **D**) Mice implanted with HSC-3 or HSC-3M cells were all sacrificed at 4 weeks after API treatment, and luciferase activity was detected every week with an IVIS imaging system. Representative bioluminescent images of mice from each group are shown. **B**, **E** Quantitative analysis of the Xenogen imaging signal intensity (photons/s/cm^2^/sr) every week. *p < 0.05, ***p < 0.001. **C**, **F** Macroscopic analysis of cervical LNs from each experimental group. Cervical LNs were removed and photographed at the end of the experiments. The appearance, number, and volume of cervical LNs are shown. Data are presented as the mean ± SD. **p < 0.01, ***p < 0.001 vs. control group and ^###^*p* < 0.001, vs. ADAMTS1-overexpressing only group. **G** Representative images of IHC staining for L1CAM in cervical LNs harvested from mice injected with HSC-3/vector, HSC-3/ADAMTS1, HSC-3M/shCtrl, or HSC-3M/shADAMTS1, either treated with or without API. **H** Kaplan–Meier survival curves for mice injected with ADAMTS1-depleted HSC-3M cells, ADAMTS1-overexpressing HSC-3 cells, or their respective control cells with or without API treatment. *p* values were analyzed by the log-rank test.

To investigate whether API exerts in vivo anticancer effects via targeting ADAMTS1, HSC-3M-shCtrl-Luc and HSC-3M-shADAMTS1-Luc cells were injected into the oral cavity of NOD-SCID mice and allowed to become established for 7 days before initiating treatment. HSC-3M-shCtrl-Luc orthotopic graft mice were treated with vehicle or API through daily IP administration for 5 days/week. Effects of API administration or ADAMTS1-knockdown on tumor growth and LN metastasis are shown in Fig. 7A–C. In vivo photon emission detection revealed that both API treatment and ADAMTS1-knockdown of HSC-3M cells showed significant antitumor growth effects compared to the control group (Fig. 7A, B). Volumes of metastatic LNs in API-treated and ADAMTS1-depleted groups were similar and significantly lower compared to control mice (Fig. 7C). In contrast to the HSC-3M xenograft model, enhancement of the tumorigenic ability in the HSC-3/ADAMTS1 xenograft model was significantly reversed by the antitumor growth effect of API (Fig. 7D, E). Consistently, increases in the number and volume of cervical metastatic LNs in HSC-3/ADAMTS1 mice were also reversed under API treatment (Fig. 7F). Results of the IHC analysis revealed the upregulation of L1CAM in tumor tissues from metastatic LNs where ADAMTS1 was overexpressed and the downregulation of L1CAM in tumor tissues from metastatic LNs where ADAMTS1 was knocked down. ADAMTS1-mediated upregulation of L1CAM could be reversed by API treatment (Fig. 7G). Most importantly, a parallel study in different animal cohorts was also carried out to evaluate the impacts of API treatment and ADAMTS1 manipulation on survival rates of OSCC-implanted mice. In an HSC-3 xenograft model, we observed that mice with HSC-3/vector tumors + API and with HSC-3/ADAMTS1 tumors respectively had longer and shorter survival times compared to mice with HSC-3/vector tumors. Moreover, API treatment significantly improved survival rates of mice implanted with HSC-3/ADAMTS1 cells (Fig. 7H, left panel). In the HSC-3M xenograft model, regardless of ADAMTS1 depletion or API treatment, both significantly prolonged survival rates of mice (Fig. 7H, right panel). Taken together, these results revealed that API is a potential inhibitor of ADAMTS1 and could effectively attenuate ADAMTS1-derived in vitro invasion and in vivo LN metastasis of OSCC.

## Discussion

To the present, metastasis and therapeutic resistance are still major causes of failure of OSCC treatment [33], and new therapeutic strategies for OSCC are urgently needed. In tumor metastasis, acquisition of the capacity to invade through the peritumoral ECM barrier is required. ADAMTS1 is one kind of ECM protease which exerts diverse impacts on the progression of different cancer types. Conflicting reports have shown both upregulation and downregulation of ADAMTS1 in different cancer types compared to normal tissues, implying both pro- and anti-tumorigenic activities of ADAMTS1 [9]. Even now, the role of ADAMTS1 in OSCC progression and its related mechanisms are still poorly understood.

Herein, we found that ADAMTS1 was downregulated in HNSCC and OSCC tissues compared to their respective normal tissues. However, ADAMTS1 expression was correlated with a poor prognosis of HNSCC patients and promoted in vitro invasion and in vivo LN metastasis of OSCC. Intriguingly, ADAMTS1 was also shown to promote tumor growth in a xenograft model, but did not influence in vitro growth rates of OSCC cells, suggesting that ADAMTS1 might indirectly promote oral tumorigenesis in vivo. Similar to our study, ADAMTS1 was also poorly expressed in hepatocellular carcinoma (HCC) compared to normal liver tissues, but did play a critical role in indirectly promoting hepatic tumorigenesis through aggravating hepatic fibrogenesis, where it was produced in abundance [34]. The functional role of ADAMTS1 in hepatic fibrosis was attributed to interacting with latent TGF-β to induce cleavage and activation of TGF-β in hepatic stellate cells (HSCs) [35], because activated TGF-β is the main cytokine that triggers differentiation of HSCs to matrix-producing myofibroblasts [36]. Oral submucosal fibrosis (OSF) is a premalignant fibrotic oral disease that is closely related to OSCC occurrence [37]. Previous studies indicated that biologically active TGF-β was upregulated in OSF tissues and promoted OSF progression [38, 39]. Whether ADAMTS1 also facilitates TGF-β activation in OSF to accelerate oral tumorigenesis should be further investigated in the future.

Although diminished ADAMTS1 expression frequently occurs within many primary cancers, progression toward metastatic disease is often associated with increased ADAMTS1. For example, primary tumors of the gastrointestinal tract and pancreas all expressed lower ADAMTS1 compared to their respective normal tissues. In contrast, matched LN metastases of gastric tumors expressed significantly higher ADAMTS1 than did primary tumors [40] and related high levels of ADAMTS1 in pancreatic tumors were also correlated with higher incidences of LN metastasis [41]. Despite the downregulation of ADAMTS1 in OSCC tissues, we also observed that high ADAMTS1 levels were correlated with high invasive abilities and cervical LN metastasis of OSCC cells. Similar to our findings, Demircan et al. reported that ADAMTS1 mRNA levels were lower in head and neck tumor samples compared to normal tissue. However, they also observed that the expression levels of ADAMTS1 were higher in the metastatic foci than in their corresponding primary tumors [42]. These results all suggest a binary role of ADAMTS1 in regulating OSCC development and progression. In contrast to the conflicting role of ADAMTS1 in regulating tumor growth in different cancer types, investigations so far into the role of ADAMTS1 in cancer metastasis promotion have consistently associated increased cell motility with upregulated ADAMTS1 [9]. ADAMTS1 as an ECM-degrading protease that was shown to indirectly induce its prometastatic effects by cleaving its substrates such as proteoglycans in the ECM and liberating cancer cells from structural barriers. For example, versican, one of the ADAMTS1 substrates, was reported to promote invasive phenotypes of gastric [43] and ovarian [44] cancer cells. In our study, we observed that knockdown of an another proteoglycan, L1CAM, significantly reversed ADAMTS1-induced invasion of OSCC cells, implying that L1CAM plays a critical role in the ADAMTS1-mediated invasive phenotype in OSCC cells. L1CAM is a 200~220-kDa (L1-200-220) transmembrane glycoprotein, and its overexpression was shown to induce invasion, metastasis, stemness, and chemoresistance in several solid tumors such as ovarian, endometrial, and pancreatic cancers [24, 45]. L1CAM was also reported to be cleaved by ADAM family proteinases and to exert its functions as a soluble form released from the cell surface. For example, L1-200 and L1-80 are substrates of the ADAM10 protease, which generates a membrane-bound 32-kDa (L1-32) and a soluble 180-kDa (L1-180) fragment [24]. Our study showed that overexpression and knockdown of ADAMTS1 respectively induced upregulation and downregulation of L1-180 in OSCC cells, Moreover, the secreted L1CAM level was also affected in ADAMTS1-manipulated OSCC cells. Furthermore, treatment with the soluble recombinant L1CAM protein (rhL1CAM) actually increased the invasive ability of OSCC cells in the present and previous studies [46]. We also observed that ADAMTS1 physically interacts with L1CAM, suggesting that ADAMTS1 might bind with L1CAM to cleave L1CAM, generate soluble L1-180, and promote invasion and LN metastasis in OSCC cells. In addition, previous studies indicated that cleaved fragments of Reelin were binding partners of L1CAM that mediated cleavage of L1CAM, and Reelin itself was reported to be cleaved by the ADAMTS family [47, 48]. Whether ADAMTS1 can directly cleave L1CAM or indirectly by cleaving L1CAM’s binding partner needs to be further investigated in the future.

To further dissect downstream effectors of the ADAMTS1-L1CAM axis to promote OSCC cell invasion, the STRING database showed that EGFR may be a potential interactor with L1CAM. Actually, Donier et al. indicated that L1CAM was observed to interact with EGFR when L1CAM and EGFR were overexpressed in HEK293 cells [28]. Herein, we also observed the interaction of EGFR with L1CAM and ADAMTS1 when ADAMTS1, L1CAM, and EGFR were overexpressed in HEK293 cells. Moreover, we found that the ADAMTS1-L1CAM axis could activate EGFR and its downstream signals such as Src, Akt, and Stat3 to further promote the invasive phenotype in OSCC cells. Previously, ADAMTS1 was reported to promote metastasis of murine breast cancer through activating EGFR by shedding HB-EGF and amphiregulin [49]. Our present results suggested that L1CAM is another downstream effector of ADAMTS1 that regulates EGFR activity and cancer progression. In addition to EGFR activation, we surprisingly found that the total EGFR level was also modulated by the ADAMTS1-L1CAM axis. Moreover, EGFR-knockdown in ADAMTS1-overexpressing HSC-3 cells dramatically reversed upregulation of ADAMTS1 and L1CAM. Taken together, our results indicated that the ADAMTS1-L1CAM axis not only increases EGFR activity but also EGFR expression, and EGFR-activated signaling may exert positive feedback regulation on ADAMTS1 expression. The cyclic increase in the ADAMTS1-L1CAM axis and EGFR activation led to an exacerbation of the invasive abilities of OSCC cells. However, how L1CAM promotes EGFR activation and expression in OSCC cells needs to be further investigated. For example, L1CAM was reported to promote activation of EGFR signaling through Cis-interactions in *Drosophila* S2 cells [27], which suggested that L1CAM might activate EGFR signaling in OSCC cells through directly binding of EGFR. In addition, L1CAM-integrin binding was shown to induce interleukin (IL)-1β production in pancreatic cancers [50], and IL-1β was reported to transactivate EGFR in OSCC [51], implying that the interaction of L1CAM and integrin might promote EGFR activation via inducing IL-1β upregulation in OSCC. All of these issues mentioned above need to be further determined in the future.

Our GSEA analysis showed that the EMT was the top Hallmark gene set among HNSCC patients with high expression levels of ADAMTS1, L1CAM, or EGFR. In addition to latent TGF-β cleavage by ADAMTS1 in HSCs mentioned above, ADAMTS1 was reported to promote the EMT and invasion via inducing upregulation of TGF-β protein levels in lung cancer cells [52]. Actually, we also observed that overexpression and knockdown of ADAMTS1 respectively induced an increase and decrease of cleaved TGF-β in OSCC cells (Supplementary Fig. 7). Moreover, TGF-β was shown to upregulate L1CAM to trigger binding of integrins, resulting in induction of IL-1β secretion and EMT promotion in pancreatic and breast cancers [53, 54]. In pancreatic cancer, EGF signaling triggered the EMT via integrin/EGFR/MAPK signaling, which induces decreases in E-cadherin and ZO-1 [55]. In OSCC, EGFR overexpression was correlated with EMT-mediated cancer metastasis [29]. Our present results showed that ADAMTS1 overexpression and knockdown in OSCC cells respectively led to EMT promotion and inhibition, as evidenced by downregulating and upregulating of ZO-1 and E-cadherin. Knockdown of L1CAM or EGFR can reverse the ADAMTS1 overexpression-induced EMT. Summarizing these observations, we suggest that the ADAMTS1-L1CAM-EGFR axis can induce the EMT to promote aggressiveness of OSCC cells. Further studies are needed to investigate the roles of TGF-β, integrins, and IL-1β in the ADAMTS1-L1CAM-EGFR axis-mediated progression of OSCC cells.

Recently, API was used as a traditional medicine for its anticancer activities and low toxicity toward normal cells. Accumulating evidence indicates that API exhibits antimetastatic activity in many cancers via suppressing the EMT or modulating ECM-degrading enzymes [56]. Until now, little information about the effects of API on the invasiveness and metastasis of OSCC cells is available. API was shown to suppress expressions of ADAMTS4 and ADAMTS5 in chondrocytes [31]. Herein, we found for the first time that API is a potential inhibitor of the ADAMTS1-L1CAM-EGFR axis and can effectively attenuate ADAMTS1-induced increases of in vitro invasion and in vivo LN metastasis of OSCC. API was shown to reduce levels of p-EGFR, p-Akt, and p-Stat3 in nasopharyngeal carcinoma [57]. Moreover, API was also reported to induce apoptosis of HNSCC cells by impairing EGFR/ErbB2 signaling [58]. Our present study showed that EGFR-activated signaling may regulate ADAMTS1 expression, which suggests that the inhibitory effect of API on ADAMTS1 in OSCC cells might be due to inhibition of EGFR activating signals.

**Fig. 8:**
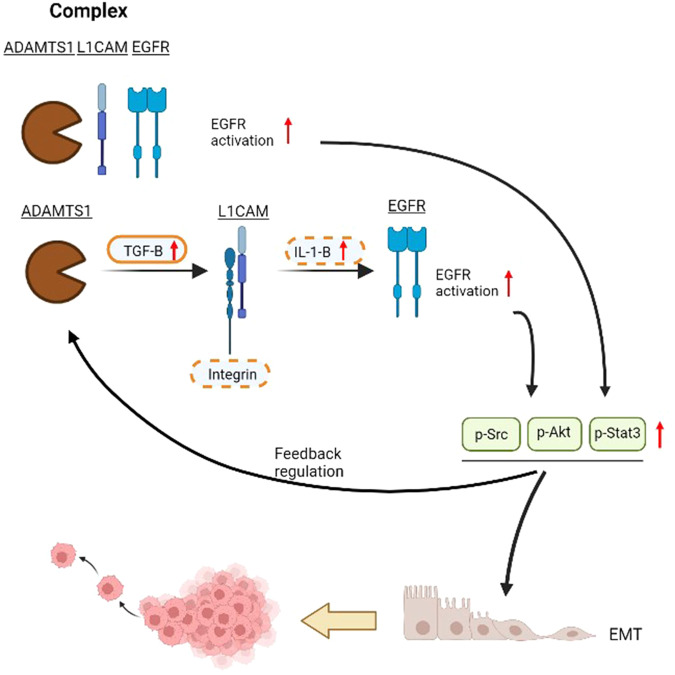
Schematic presentation depicting the ADAMTS1-L1CAM-EGFR axis in promoting the epithelial-mesenchymal transition (EMT) and metastasis of oral squamous cell carcinoma (OSCC). EGFR activation might be triggered by formation of the ADAMTS1-L1CAM-EGFR complex or through ADAMTS1-mediated TGF-β upregulation to subsequently induce L1CAM upregulation and L1CAM-integrin binding, resulting in induction of IL-1β secretion. Bold dashed ovals indicate hypothetical molecules that participate in the ADAMTS1-L1CAM axis to transactivate EGFR signaling, and EGFR-activated signaling may exert positive feedback regulation on ADAMTS1 expression. Cyclic increases in ADAMTS1 and EGFR activation lead to exacerbation of the EMT and invasive abilities of OSCC cells.

In this study, we provided a novel mechanism to address how ADAMTS1 regulates tumor metastasis in OSCC (Fig. 8). ADAMTS1 acts as a tumor metastasis promoter in OSCC, and L1CAM and EGFR are critical determinants regulated by ADAMTS1 when executing its prometastatic effect via EMT induction. In the clinic, the poor prognostic effects of ADAMTS1, L1CAM, and EGFR were observed only in HNSCC among 33 different cancer types suggesting that the ADAMTS1-L1CAM-EGFR axis plays a specific role in regulating OSCC progression. Our findings can promote a better understanding of the mechanisms of metastasis; we also discovered API as a potential therapeutic agent for managing OSCC metastasis via targeting ADAMTS1.

### Supplementary information

Supplementary data

Reproducibility checklist

Original Data File

## Data Availability

The experimental data generated and analyzed in this current study are available from the corresponding author upon reasonable request.
