# Supplementary material for: Cyclic increase in the ADAMTS1-L1CAM-EGFR axis promotes the EMT and cervical lymph node metastasis of oral squamous cell carcinoma
Source: Cell Death Dis. 2024 Jan 23;15(1):82. doi: 10.1038/s41419-024-06452-9 (PMC10805752; doi:10.1038/s41419-024-06452-9)
Supplement: Supplementary file 1 — Supplementary data [file 41419_2024_6452_MOESM1_ESM.docx]

**Supplemental Information**

**Title:**

**Cyclic increase in the ADAMTS1-L1CAM-EGFR axis promotes the EMT and cervical lymph node metastasis of oral squamous cell carcinoma**

Ming-Hsien Chien, Yi-Chieh Yang, Kuo-Hao Ho, Yi-Fang Ding, Li-Hsin Chen, Wen-Kuan Chiu, Ji-Qing Chen, Min-Che Tung, Michael Hsiao, and Wei-Jiunn Lee

Correspondence to: Dr. Wei-Jiunn Lee (E-mail: wjlee@tmu.edu.tw)

**Figure Legends**

**
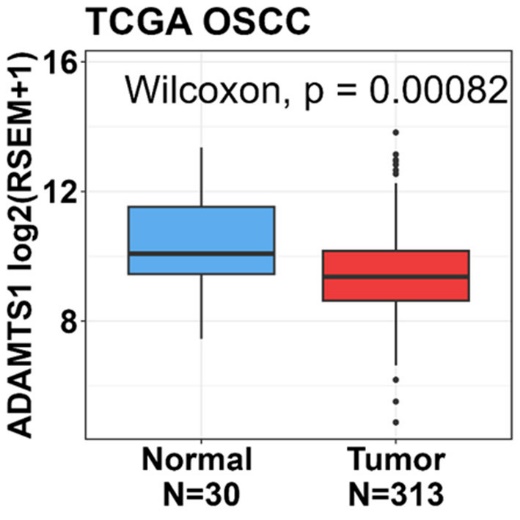
**

**
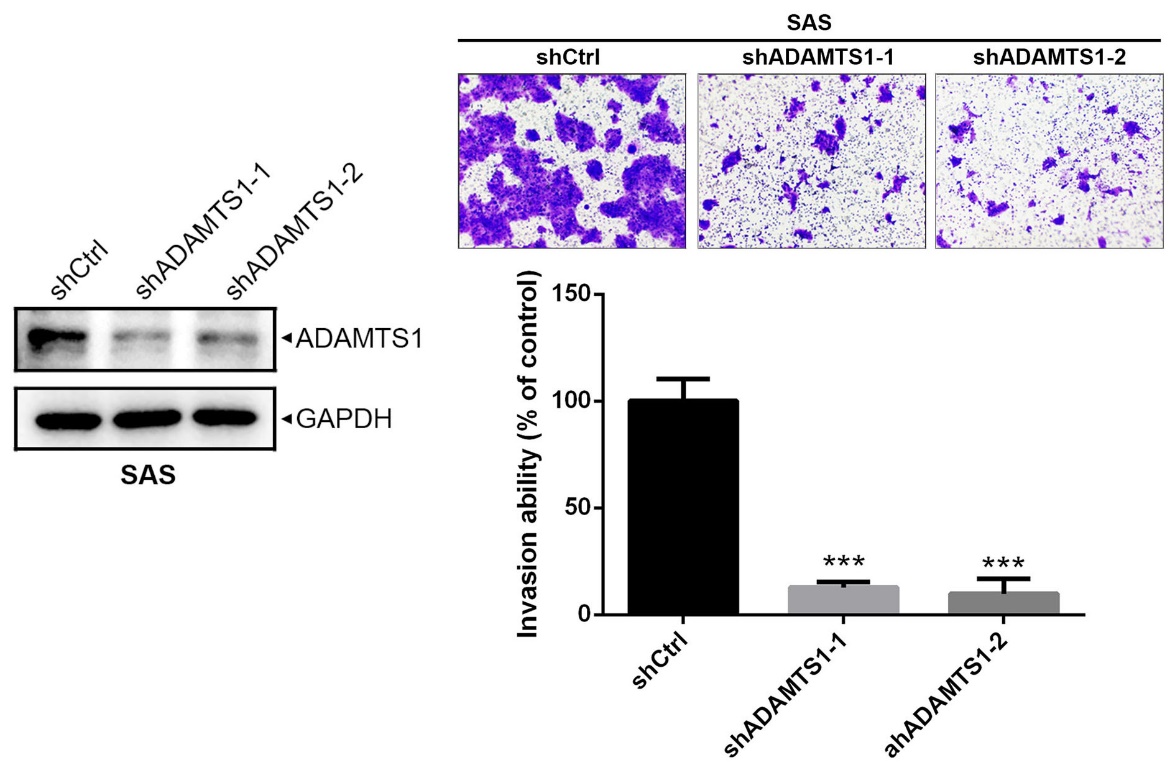
Figure S1. ADAMTS1 expression levels are significantly lower in oral squama cell carcinoma (OSCC) tissues compared to normal tissues.** *ADAMTS1* transcripts in normal and OSCC tissues (tumors occurred on buccal mucosa, alveolar ridge, floor of mouth, hard palate, oral cavity, and oral tongue) were analyzed using data from TCGA-head-neck squamous cell carcinoma. Statistical significance was analyzed by a Wilcoxon signed-rank test.

**Figure S2. Knockdown of ADAMTS1 inhibits invasive abilities of SAS cells.** Left panel, Western blot analysis of ADAMTS1 expressions in SAS cells ADAMTS1 shRNAs. Right panel, a Matrigel-invasion assay was performed to determine the invasive abilities of SAS carrying ADAMTS1 shRNAs or shControl (shCtrl). Quantitative results by counting invaded cells in a 100× field. Multiples of differences are presented as the mean ± SD of three independent experiments. *** *P*< 0.001, compared to the control group.


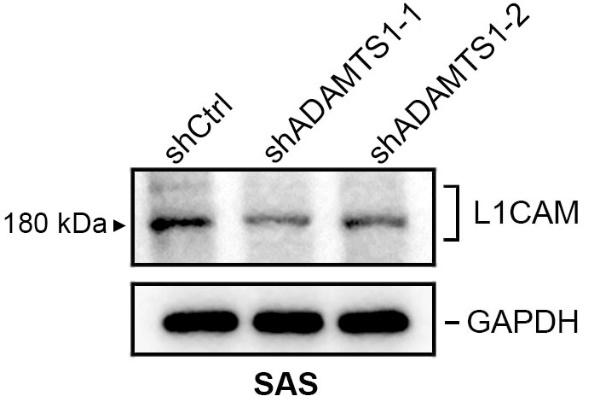


**Figure S3. Effects of ADAMTS1 knockdown on L1CAM expression in SAS cells.** SAS cells were infected with a lentivirus carrying either two specific ADAMTS1 shRNAs or shControl (shCtrl) and subjected to a Western blot analysis to determine expressions of L1CAM. Quantitative results of L1CAM proteins were adjusted to GAPDH protein levels.

**
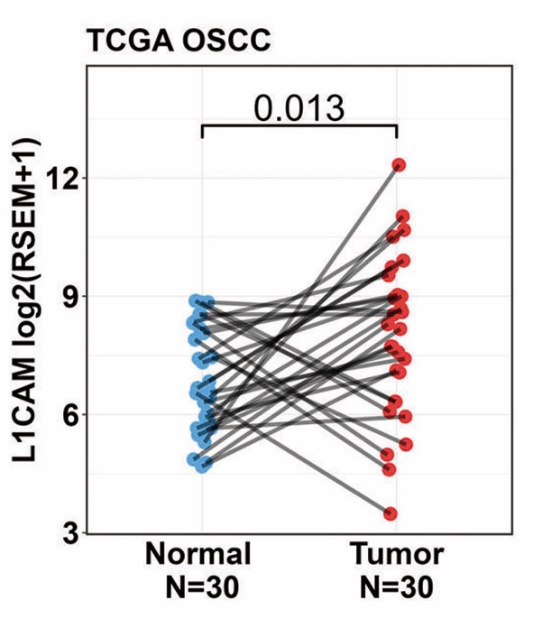
**

**Figure S4. Expression levels of L1CAM in oral squama cell carcinoma (OSCC) tissues and their matched normal tissues.** L1CAM expression was analyzed in 30 matched OSCC (tumors occurred on buccal mucosa, alveolar ridge, floor of mouth, hard palate, oral cavity, and oral tongue) tissues and their corresponding normal tissues using data from TCGA.


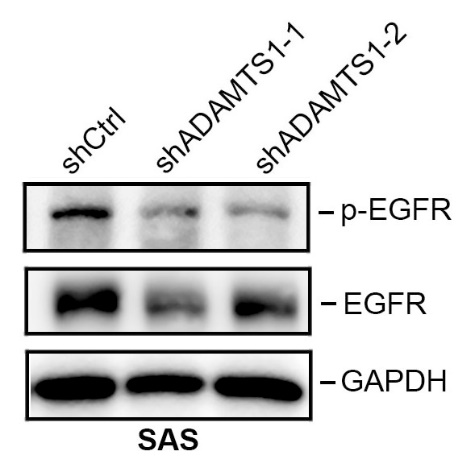


**Figure S5.** **Effects of ADAMTS1 knockdown on activation and expression of EGFR in SAS cells.** SAS cells were infected with a lentivirus carrying either two specific ADAMTS1 shRNAs or shControl (shCtrl) and subjected to a Western blot analysis to determine the levels of p-EGFR and EGFR. Quantitative results of EGFR proteins were adjusted to GAPDH protein levels.


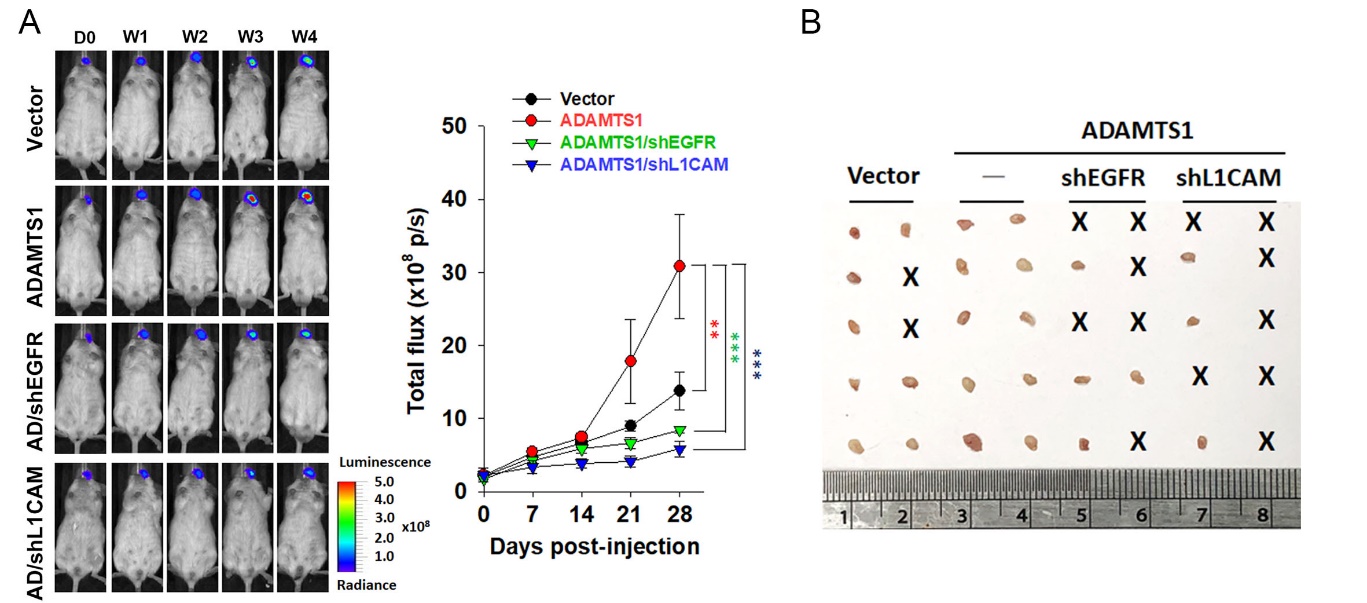


**Figure S6** **ADAMTS1 promotes the tumorigenic and metastatic potential of HSC-3 cells by modulating signals associated with EGFR and L1CAM in vivo.** (A) In the left panel, male NOD/SCID mice were orthotopically injected with luciferase-tagged HSC-3 cells overexpressing ADAMTS1, HSC-3 cells overexpressing ADAMTS1 with EGFR depletion, or HSC-3 cells overexpressing ADAMTS1 with L1CAM depletion. Whole-body bioluminescence imaging was performed weekly for four weeks after cell injection. The right panel displays the quantitative analysis of Xenogen imaging signal intensity (photons/s/cm^2^/sr) at each weekly time point. ***p* < 0.01, ****p* < 0.001. (B) Macroscopic analysis of the cervical lymph nodes (LNs) was conducted. The appearance and enumeration of cervical LNs were documented following their removal.

**
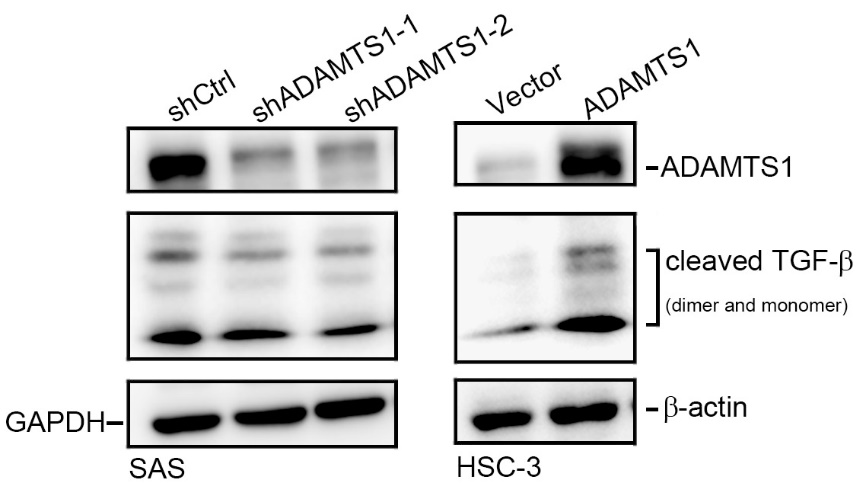
**

**Figure S7. ADAMTS1 expression promotes the expression of active transforming growth factor-beta (TGF-β) in oral squama cell carcinoma (OSCC) cells.** A Western blot analysis of active TGF-β (cleaved TGF-β) expressions in SAS (left) and HSC-3 (right) cells respectively expressing ADAMTS1 shRNAs and ADAMTS1-flag.
