# Supplementary material for: Cyclic increase in the ADAMTS1-L1CAM-EGFR axis promotes the EMT and cervical lymph node metastasis of oral squamous cell carcinoma
Source: Cell Death Dis. 2024 Jan 23;15(1):82. doi: 10.1038/s41419-024-06452-9 (PMC10805752; doi:10.1038/s41419-024-06452-9)
Supplement: Supplementary file 3 — Original Data File [file 41419_2024_6452_MOESM3_ESM.pdf]

Figure 1C

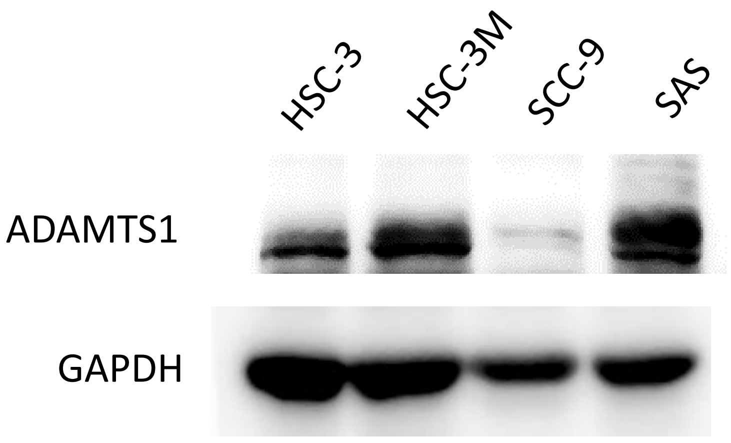

Figure 1E

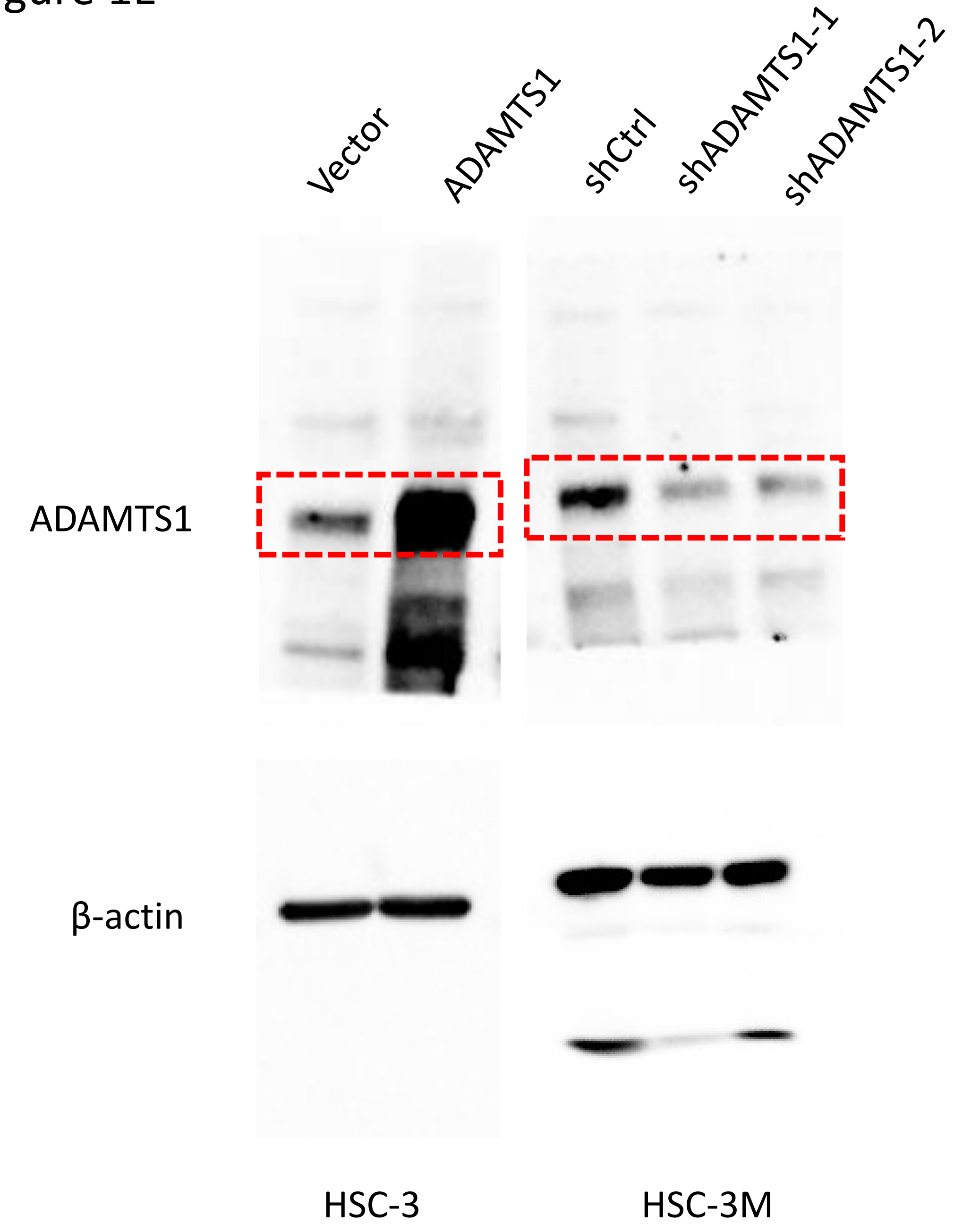

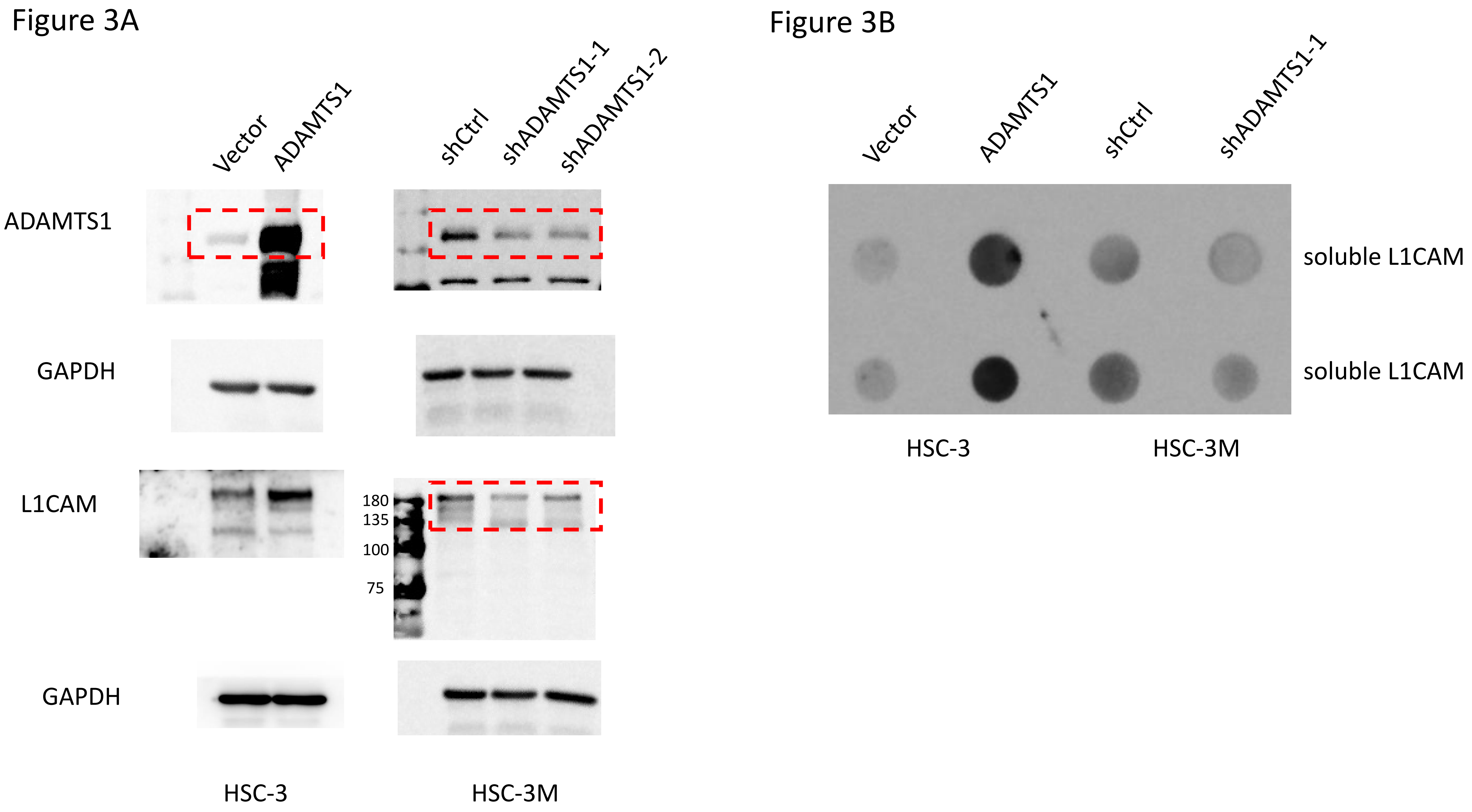

Figure 3A
Vector
ADAMTS1
shCtrl
shADAMTS1-1
shADAMTS1-2
ADAMTS1
GAPDH
L1CAM
180
135
100
75
GAPDH
HSC-3
HSC-3M
Figure 3B
Vector
ADAMTS1
shCtrl
shADAMTS1-1
soluble L1CAM
soluble L1CAM
HSC-3
HSC-3M

Figure 4B

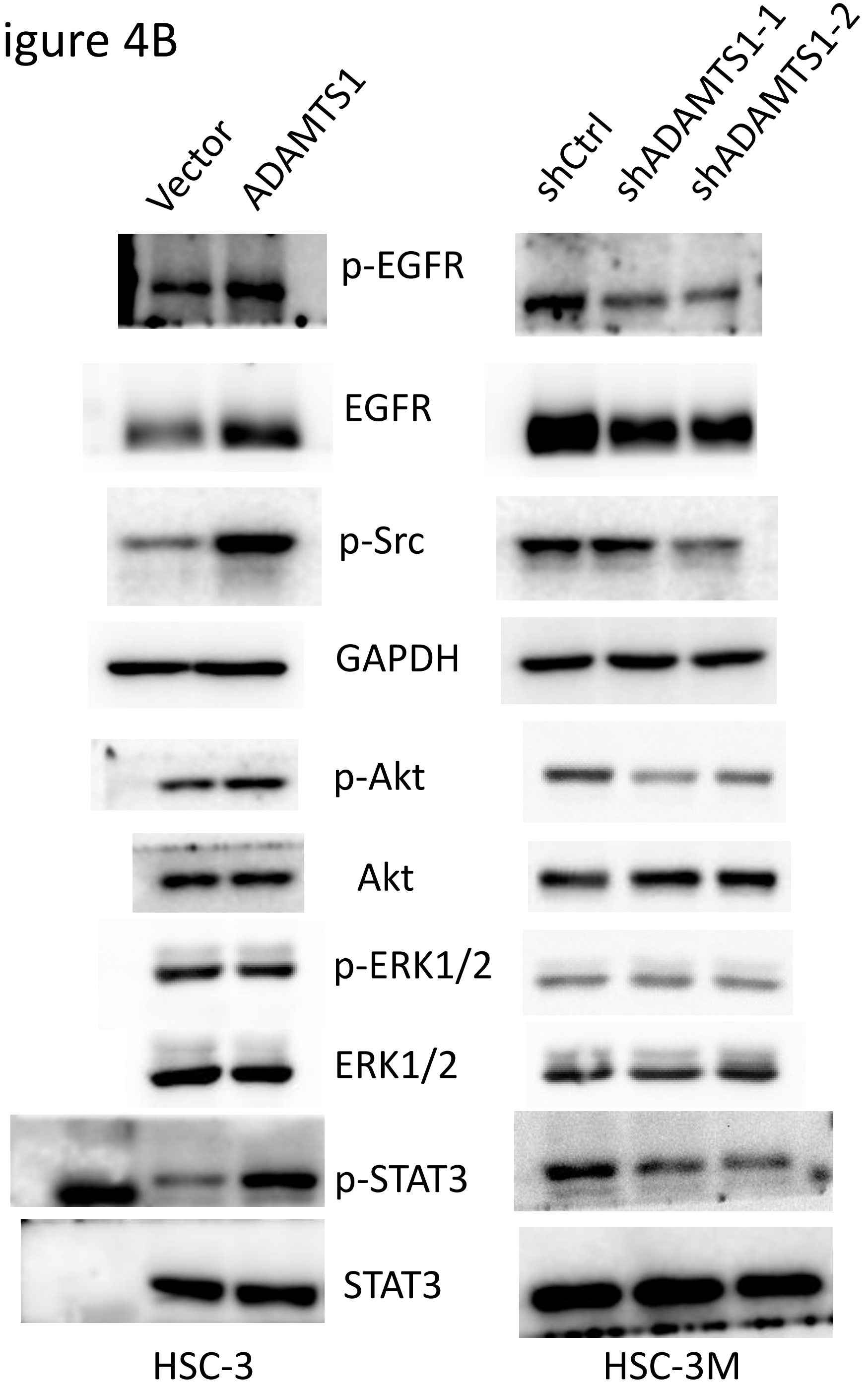

Figure 4D

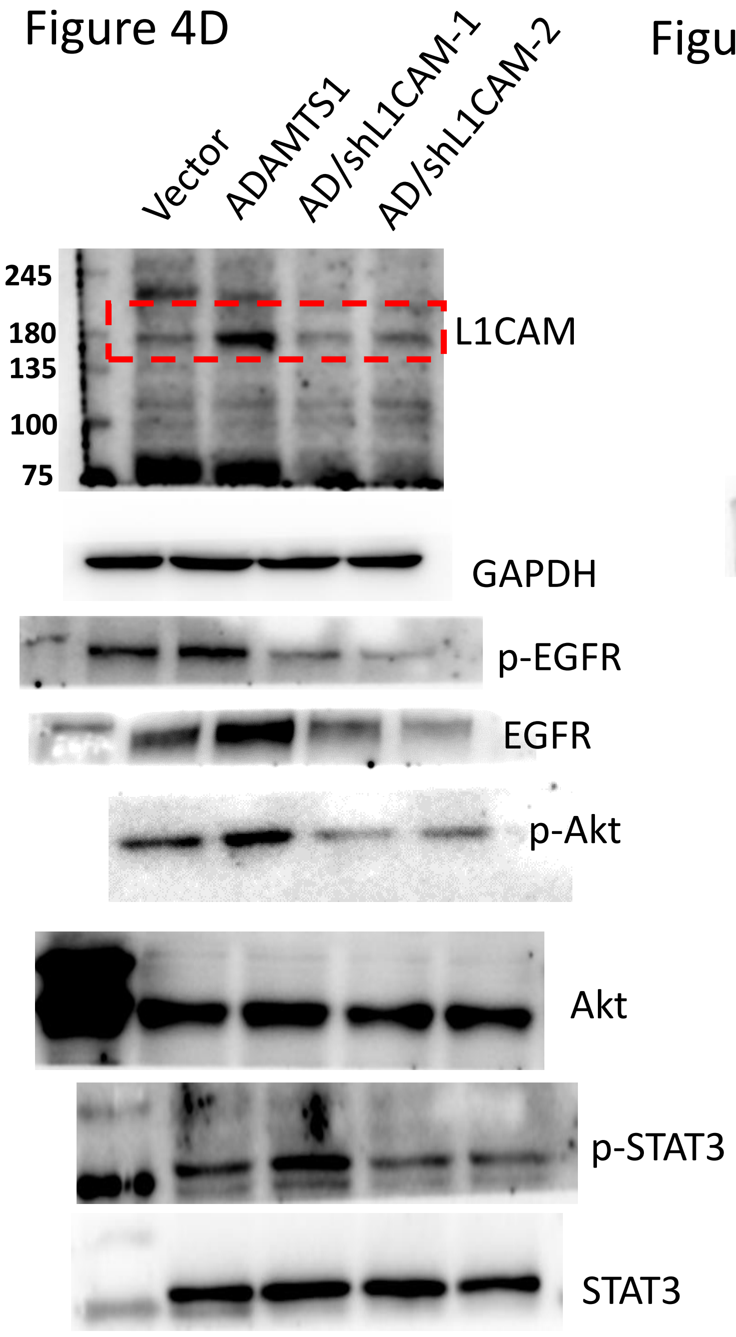

Figure 4E

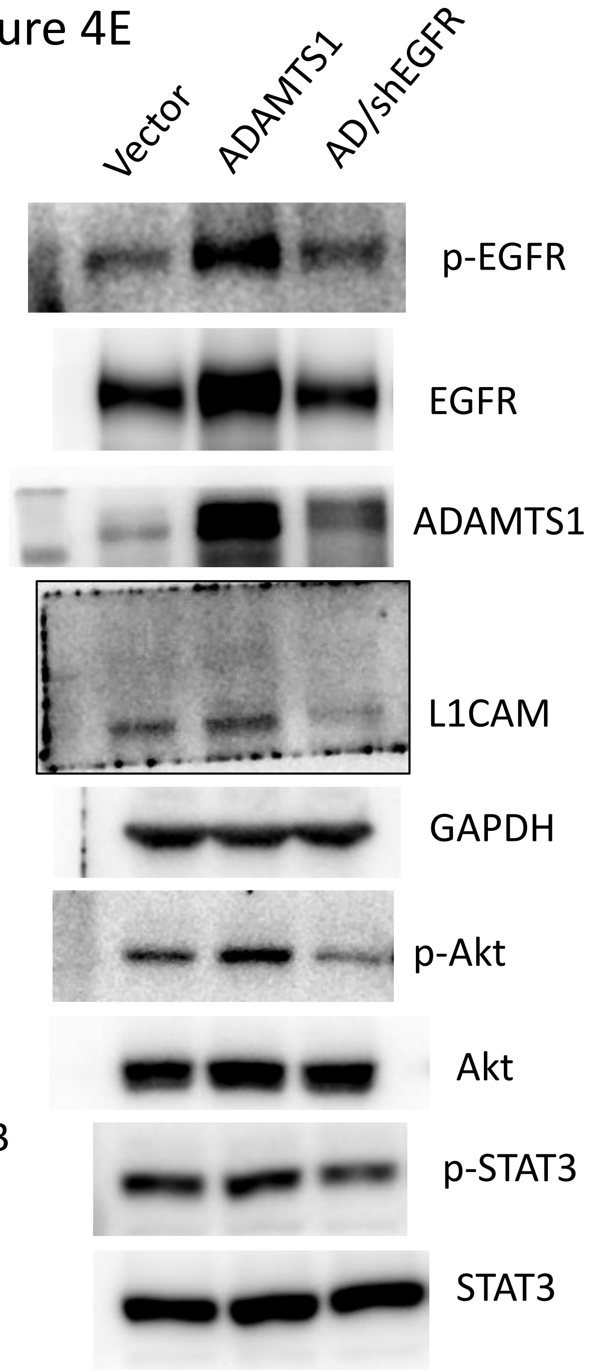

Figure 4F

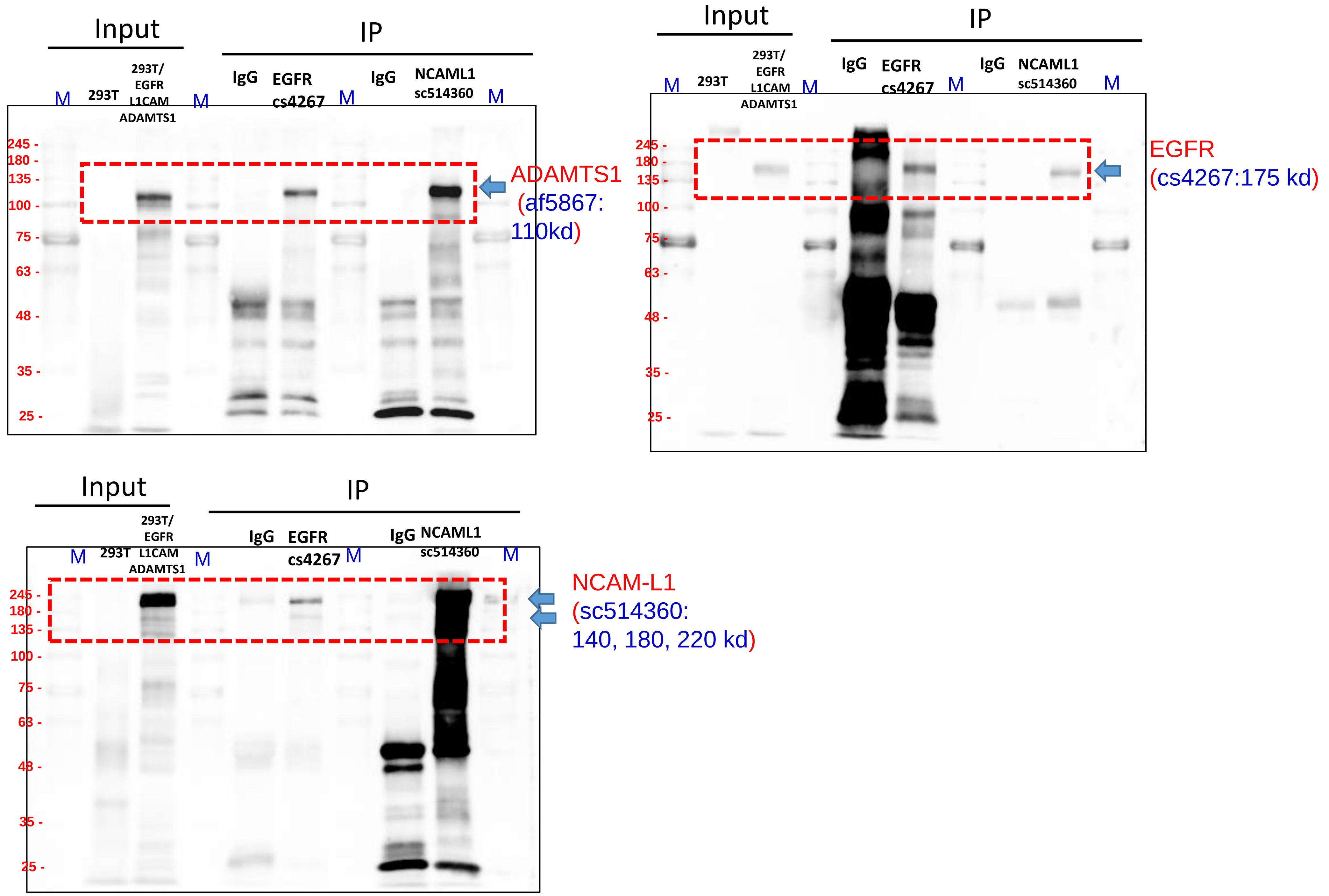

Input
IP
M
293T
293T/ EGFR L1CAM ADAMTS1
IgG
EGFR cs4267
NCAML1 sc514360
245 -
180 -
135 -
100 -
75 -
63 -
48 -
35 -
25 -
ADAMTS1
(af5867: 110kd)
EGFR
(cs4267:175 kd)
NCAM-L1
(sc514360: 140, 180, 220 kd)

Figure 5B

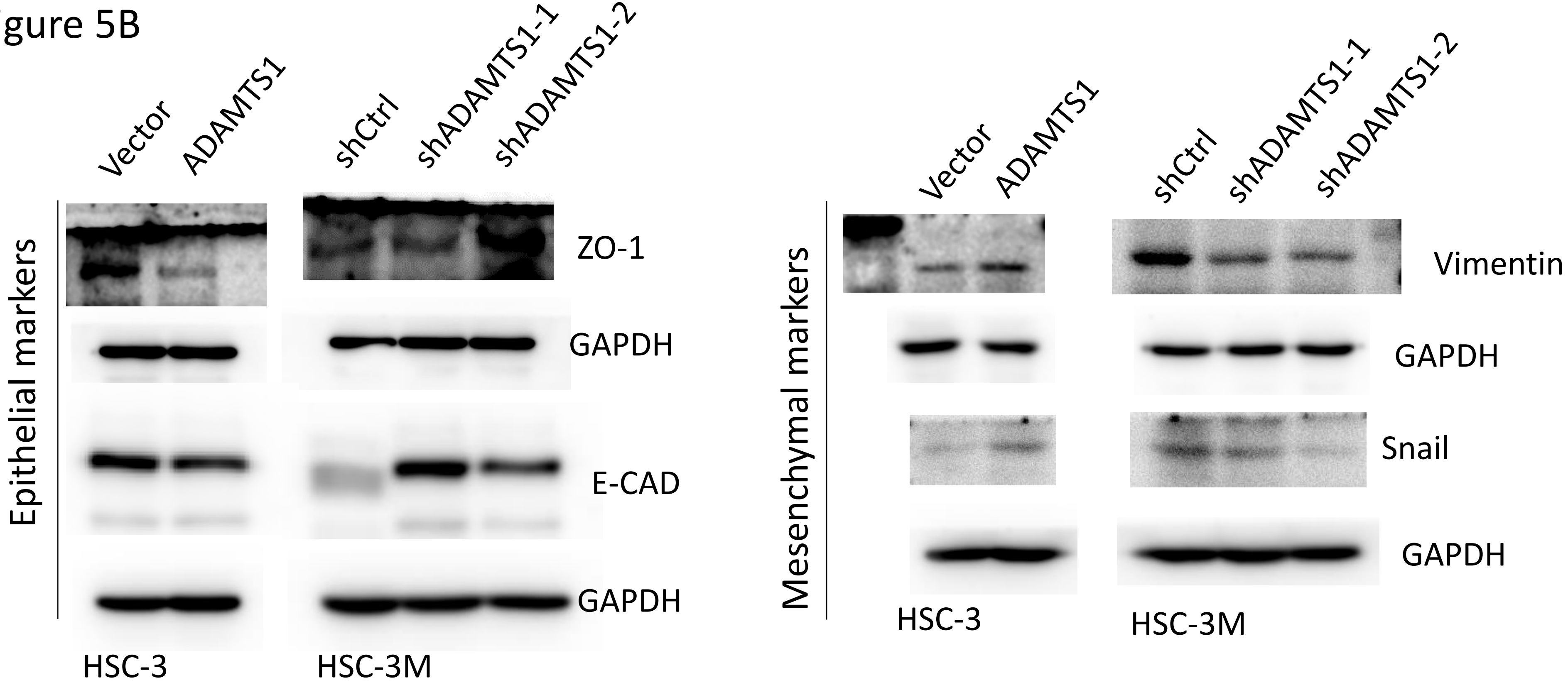

Figure 5C

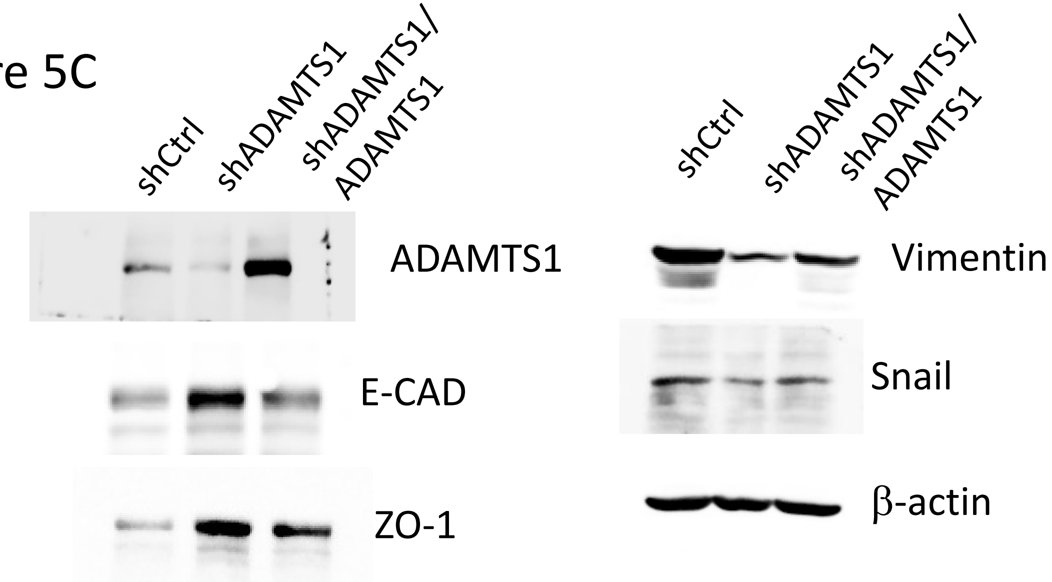

Figure 5D

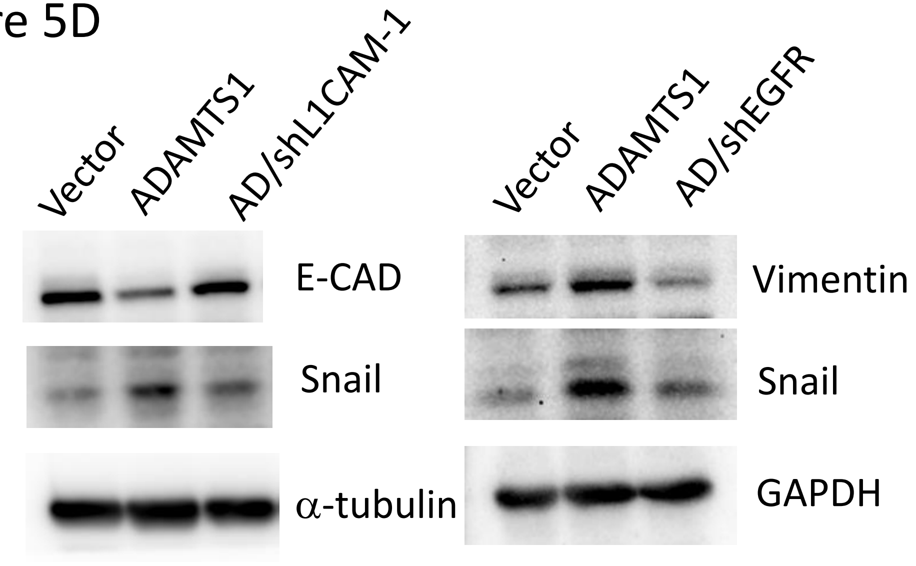

Figure 6A

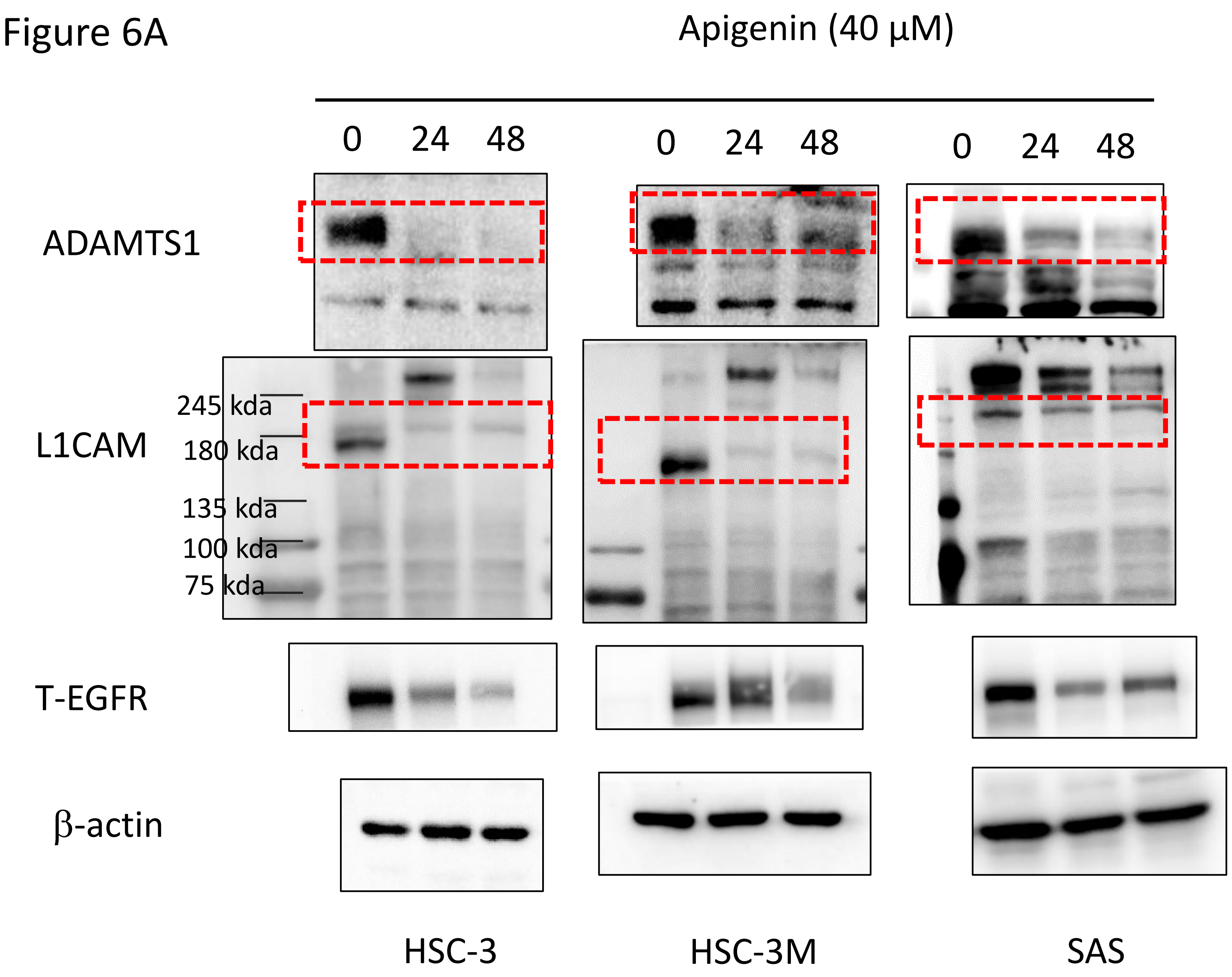

Figure 6C

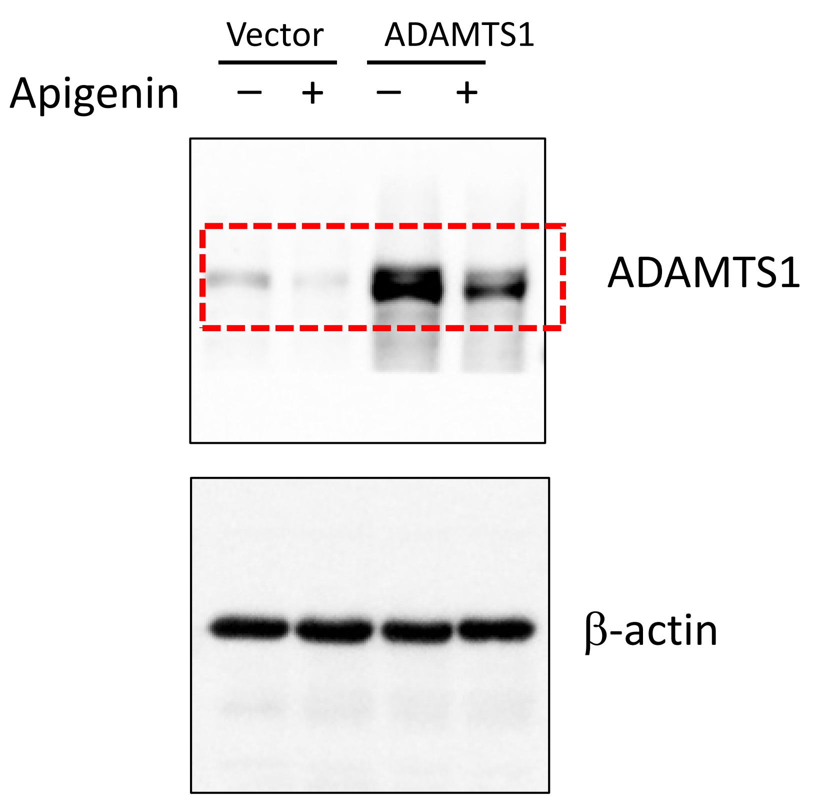

Figure S2

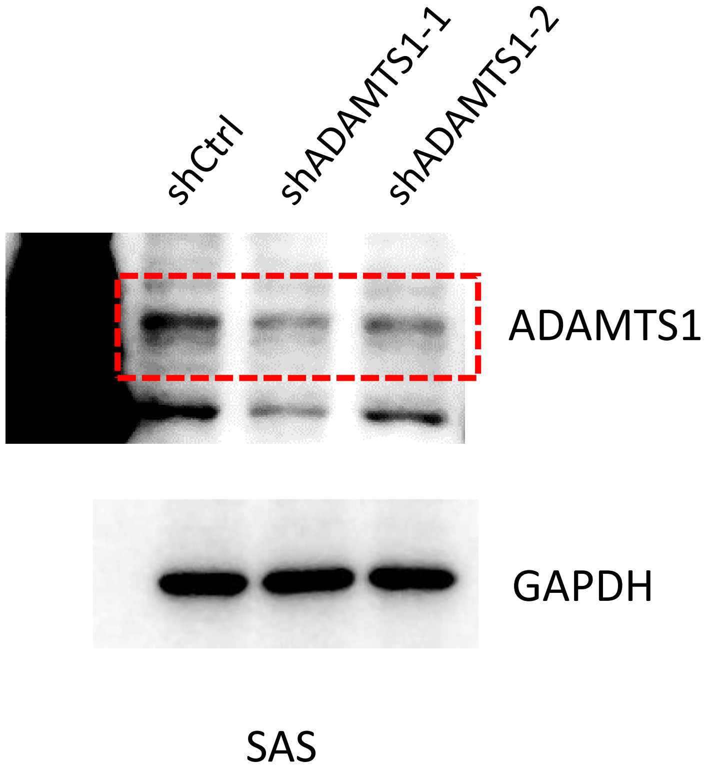

Figure S3

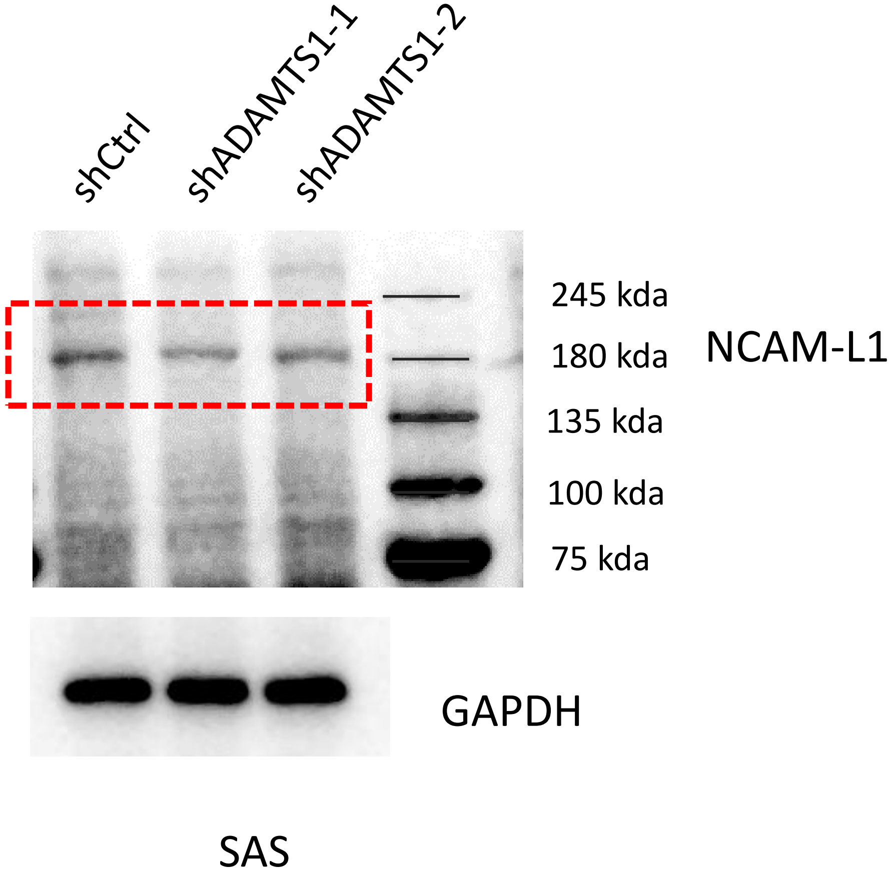

Figure S5

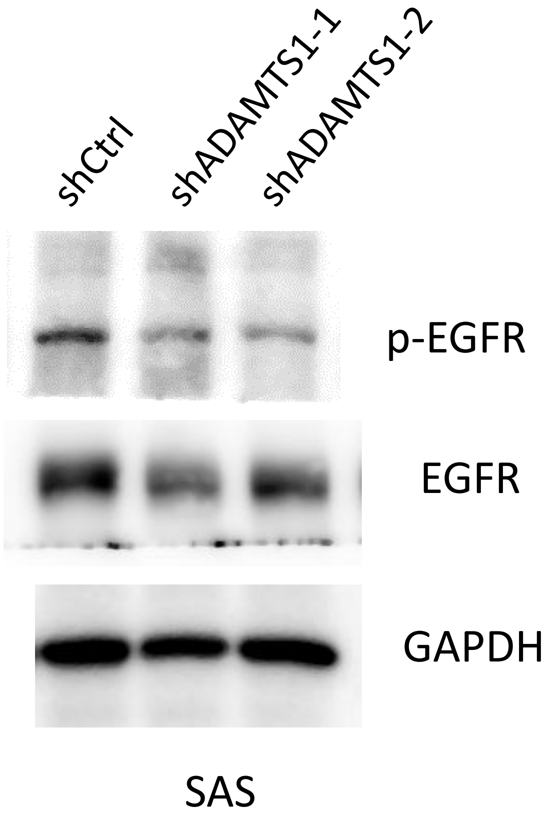

Figure S7

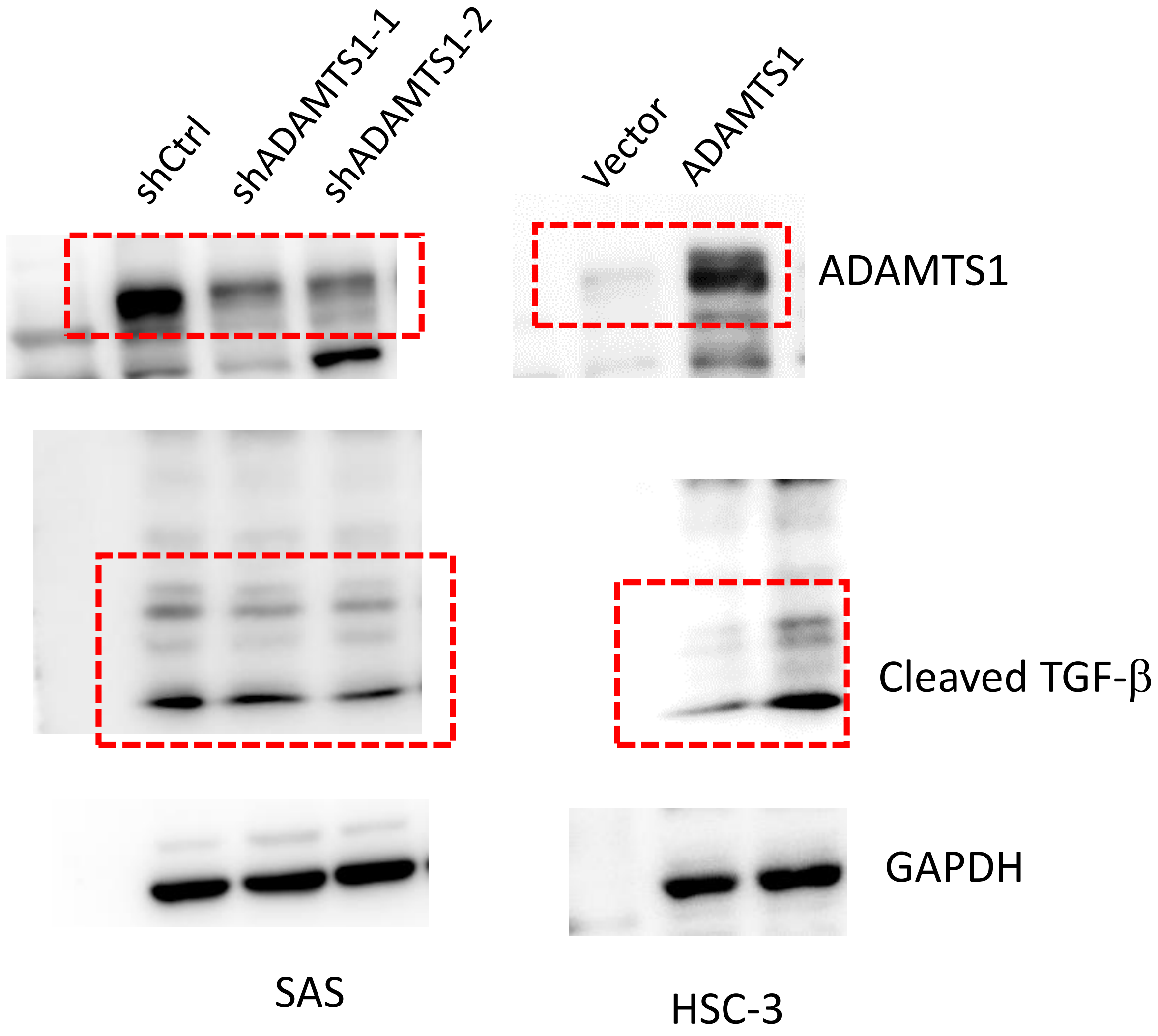
shCtrl
shADAMTS1-1
shADAMTS1-2
Vector
ADAMTS1
ADAMTS1
Cleaved TGF-β
GAPDH
SAS
HSC-3
